# The ancestral type of the R-RAS protein has oncogenic potential

**DOI:** 10.1186/s11658-024-00546-0

**Published:** 2024-02-21

**Authors:** Antea Talajić, Kristina Dominko, Marija Lončarić, Andreja Ambriović-Ristov, Helena Ćetković

**Affiliations:** 1 Laboratory for Molecular Genetics, Division of Molecular Biology, Ruđer Bošković Institute, 10000 Zagreb, Croatia; 2 Laboratory for Cell Biology and Signalling, Division of Molecular Biology, Ruđer Bošković Institute, 10000 Zagreb, Croatia

**Keywords:** Cancer, Cell migration, Cell proliferation, Evolution, Focal adhesion, Intracellular localization, Metazoa, R-RAS2, Small GTPase, Porifera

## Abstract

**Background:**

The R-RAS2 is a small GTPase highly similar to classical RAS proteins at the regulatory and signaling levels. The high evolutionary conservation of R-RAS2, its links to basic cellular processes and its role in cancer, make R-RAS2 an interesting research topic. To elucidate the evolutionary history of R-RAS proteins, we investigated and compared structural and functional properties of ancestral type R-RAS protein with human R-RAS2.

**Methods:**

Bioinformatics analysis were used to elucidate the evolution of R-RAS proteins. Intrinsic GTPase activity of purified human and sponge proteins was analyzed with GTPase-Glo^TM^ Assay kit. The cell model consisted of human breast cancer cell lines MCF-7 and MDA-MB-231 transiently transfected with EsuRRAS2-like or HsaRRAS2. Biological characterization of R-RAS2 proteins was performed by Western blot on whole cell lysates or cell adhesion protein isolates, immunofluorescence and confocal microscopy, MTT test, colony formation assay, wound healing and Boyden chamber migration assays.

**Results:**

We found that the single sponge R-RAS2-like gene/protein probably reflects the properties of the ancestral R-RAS protein that existed prior to duplications during the transition to Bilateria, and to Vertebrata. Biochemical characterization of sponge and human R-RAS2 showed that they have the same intrinsic GTPase activity and RNA binding properties. By testing cell proliferation, migration and colony forming efficiency in MDA-MB-231 human breast cancer cells, we showed that the ancestral type of the R-RAS protein, sponge R-RAS2-like, enhances their oncogenic potential, similar to human R-RAS2. In addition, sponge and human R-RAS2 were not found in focal adhesions, but both homologs play a role in their regulation by increasing talin1 and vinculin.

**Conclusions:**

This study suggests that the ancestor of all animals possessed an R-RAS2-like protein with oncogenic properties similar to evolutionarily more recent versions of the protein, even before the appearance of true tissue and the origin of tumors. Therefore, we have unraveled the evolutionary history of R-RAS2 in metazoans and improved our knowledge of R-RAS2 properties, including its structure, regulation and function.

**Supplementary Information:**

The online version contains supplementary material available at 10.1186/s11658-024-00546-0.

## Background

The Ras-related (R-Ras) subfamily, comprising R-RAS, R-RAS2/TC21, and M-RAS, is a part of the Ras superfamily of small GTPases. Ras proteins play an essential role in signal transduction pathways that control cellular processes such as proliferation, survival, migration, and differentiation. While the canonical Ras proteins (H-Ras, K-Ras, and N-Ras) have been extensively studied, the physiological and pathological functions of the R-Ras subfamily are poorly understood. However, emerging evidence highlights their importance in regulating cellular processes associated with cell morphology, adhesion, and migration [1, 2]. The R-Ras subfamily members exhibit high structural similarities, particularly in the conserved guanine nucleotide-binding domains crucial for GTPase activity. The amino acid residues essential for GTPase activity are categorized into five motifs referred to as G1, G2, G3, G4, and G5 boxes, which form the catalytic site responsible for the hydrolysis of GTP. Moreover, in addition to the G boxes, there are two other highly conserved regions partially overlapping with G2 and G3 boxes, that significantly contribute to the functional dynamics of R-RAS. These regions, namely *SwitchI* and *SwitchII* domains, are conserved across all three R-RAS subfamily members and crucial for transitioning between the activated and inactivated conformations of R-RAS proteins [2–5]. However, the R-Ras subfamily members differ in their N- and C-terminal regions. The differences within C-terminal hypervariable regions (HVR) affect their distinct localization and downstream signaling pathways [2, 3]. R-Ras subfamily proteins also differ in posttranslational modifications at their C-termini. R-RAS is geranylgeranylated and palmitoylated, while M-RAS is geranylgeranylated. R-RAS2 is palmitoylated and farnesylated, similar to the classical Ras proteins, explaining their similar subcellular localization and signaling pathways [2].

Human R-RAS protein mainly localizes to focal adhesions on the plasma membrane [6], where it regulates integrin functions by enhancing focal adhesion formation, cell adhesion, and cell spreading [7, 8]. R-RAS2/TC21 localizes to the plasma membrane and the Golgi apparatus [9, 10], suggesting its involvement in intracellular vesicle trafficking and protein secretion. Our previous study confirmed the localization of R-RAS2 in the plasma membrane and vesicular membranes, possibly the endocytic vesicles, in HeLa and MJ90 fibroblast cell lines [11]. Endogenous wild-type R-RAS2 specifically localizes to focal adhesions in cancer cell lines from different cancer types (ovary, breast, fibrosarcoma) and species (human and mouse), suggesting its role in cell adhesion [12]. M-RAS plays an important role in cytoskeletal remodeling, cell migration, osteoblastic and neuronal differentiation, dendrite formation, and lymphocyte adhesion [13, 14]. Numerous studies have shown that oncogenic mutations in R-RAS2 exhibit equal or even higher transformation capacities when compared to classic Ras proteins [15–18]. Moreover, mutations in R-RAS2 have been implicated in the induction of breast tumorigenesis and late-stage metastasis [19]. Besides oncogenic mutations, overexpression of the wild-type R-RAS2 can also induce breast cancer transformation [20]. Elevated levels of wild-type R-RAS2 are present in various cancers, including esophageal tumors [21], oral cancer [22], skin cancer [23], lymphoma [24], and chronic lymphocytic leukemia (CLL) in which R-RAS2, in addition to its role in cancer, also regulates immunological development and homeostasis, mainly via the PI3K signaling pathway [20].

In non-bilaterian animals, the R-Ras subfamily is characterized by the presence of a single homolog. However, the specific functional relationship between this ancestral type protein and the three members of the R-Ras subfamily found in the human genome remains unclear. Through comprehensive bioinformatics analysis, we identified that the R-Ras subfamily protein found in non-bilaterian animals is most closely related to the human R-RAS2 protein. Therefore, we refer to this protein as R-RAS2-like. To further investigate the functional relationships of this ancestral type protein and human R-RAS2, we analyzed the biochemical and biological properties of the R-RAS2-like protein from a non-bilaterian animal, sponge *Eunapius subterraneus*, and its human homolog. By studying these properties, we aimed to contribute to a broader understanding of the role and functions of the R-RAS2 protein in various biological processes, as well as its evolutionary conservation between sponges and humans.

## Methods

### Bioinformatics analysis

The protein sequences of the R-RAS subfamily members were acquired through a blastp search of the National Center for Biotechnology Information (NCBI) database (https://blast.ncbi.nlm.nih.gov/Blast.cgi). Our evolutionary analysis included species from selected metazoan lineages and their closest unicellular relatives with publicly available sequenced genomes. Proteins containing large insertions or deletions that could appear due to incorrect gene annotation were excluded from our phylogenetic analysis. Additional file 1 includes a list of accession numbers for R-RAS, R-RAS2, and M-RAS homologs that we used in our study. Multiple protein sequences were aligned using ClustalX 2.0 [25] with no adjustment of the default parameters. Alignment editing and shading were done using GeneDoc, Version 2.7 [26]. Conserved protein domains were identified using NCBI Conserved Domain Search (https://www.ncbi.nlm.nih.gov/Structure/cdd/wrpsb.cgi) and from the original publications [2]. An overall phylogenetic tree of the R-RAS subfamily of proteins was constructed utilizing MEGA7 software [27]. The maximum likelihood phylogenetic tree was calculated using JTT + G + I evolutionary model [28], according to results obtained by ProtTest [29]. To determine the robustness of the inferred tree topology, bootstrap analysis with 1000 replicates was performed, thereby providing support values for each internal branch. Internal branches with bootstrap values greater than 50% were considered reliable subgroups, while lower values were not shown.

To generate amino acid identity and similarity matrices from multiple sequence alignment, the Matrix Global Alignment Tool (MatGAT2.01 with BLOSUM62 scores [30]), was used. The summarized identity and similarity datasets were visualized using a heat map conducted by Morpheus (https://software.broadinstitute.org/morpheus/). For intron-mapping analysis of *r-ras* subfamily genes from selected metazoan species, nucleotide sequences containing annotated intron positions were acquired from the genomic database of the NCBI (https://www.ncbi.nlm.nih.gov/genome/). The precise position and phase of each intron were subsequently confirmed through manual verification.

### Plasmid construction

Specific primers were designed to amplify cDNAs corresponding to sponge (EsuRRAS2-like) and human R-RAS2 (HsaRRAS2) proteins. EsuRRAS2-like (accession number WDZ04215.1) was amplified from the cDNA library of *E. subterraneus*, while the human homolog was amplified from a commercially available plasmid. Both EsuRRAS2L and HsaRRAS2 were amplified, sequenced, and cloned into various vectors (pET28b, pEGFPC1, pmCherryC1, and pcDNA3.1) using specific primers and restriction enzymes listed in Additional file 2: Table S1. The resulting constructs were His-, GFP-, CHERRY-, or FLAG-tagged, depending on the specific experiment.

### Protein expression and purification

Recombinant EsuRRAS2-like and HsaRRAS2 proteins were isolated using *E. coli* strain BL21 CodonPlus (DE3) for their biochemical characterization. The cDNAs for EsuRRAS2-like and HsaRRAS2 were cloned into pET28b vectors with a histidine tag at their N-terminus and introduced into *E. coli* cells via chemical transformation. The bacterial cells were cultured in LB/Kan medium at 37 °C until reaching an optical density of 0.6–0.8 at 600 nm. To induce protein expression, 0.8 mM IPTG was added, followed by incubation at 30 °C for 3 h. After the incubation, the cells were harvested, washed, and subjected to a 30-min ice incubation in a lysis buffer containing 25 mM Tris–HCl (pH 7.5), 500 mM NaCl, 10 mM imidazole, 1 mg/mL lysozyme (Sigma-Aldrich), and a protease inhibitor cocktail (Roche Applied Science). Subsequently, the cells were disrupted using sonication at 4 °C, with 3–5 cycles of 3.5 min each, to release the protein contents. The lysate was purified by centrifugation at 13280 × g and 4 °C for 30 min, followed by filtration using a sterile 0.22 μm membrane filter. The talon resin charged with cobalt (Takara) was used to purify His-tagged recombinant proteins from the filtered solution. Proteins bound to resin were then eluted with an elution buffer containing 300 mM imidazole. Finally, eluted proteins were concentrated in a storage buffer containing 25 mM Tris–HCl (pH 7.5), 300 mM NaCl, 1 mM DTT, 1 mM EDTA, and 10% glycerol using Amicon Ultra Centrifugal 10 kDa Filters (Merck). The purity of the recombinant proteins, EsuRRAS2-like-His and HsaRRAS2-His, was evaluated using SDS–polyacrylamide gel electrophoresis (SDS-PAGE).

### Intrinsic GTPase activity

To assess the intrinsic GTPase activity of EsuRRAS2-like and HsaRRAS2, we utilized the commercially available GTPase-Glo^TM^ Assay kit (Promega) following the manufacturer's guidelines. The final reactions were carried out with a molar concentration of 1 µM GTP, as suggested in the protocol for evaluating intrinsic GTPase activity. For optimizing protein concentrations, the purified HsaRRAS2 and EsuRRAS2-like were diluted serially in a GTPase/GAP buffer containing 1 µM GTP, and the assay was performed for 120 min at room temperature (25 °C). Subsequently, 10 µL of GTPase-Glo Reagent was added to the completed GTPase reaction and incubated with shaking for 30 min at room temperature. Afterward, 20 µL of Detection Reagent was added to the reaction mixture, followed by incubation for 5–10 min at room temperature. Luminescence was measured using solid white 384-well microplates (Greiner) on the Infinite M200 plate reader (Tecan).

For evaluating the intrinsic GTPase activity of sponge and human homologs, reaction mixtures were prepared for each protein at enzyme concentration of 6.25 μM (791.875 ng), as optimized in the previous step. The reactions were conducted under the same conditions as described above. Amounts and corresponding concentrations of HsaRRAS2 and EsuRRAS2-like proteins used for GTPase activity assay are shown in Additional file 2: Tables S2 and S3.

### RNA binding assay

To determine the nonspecific RNA binding ability of R-RAS2 proteins, an in vitro RNA binding assay was performed. For this purpose, the proteins of interest (EsuRRAS2-like and HsaRRAS2) were incubated with polyuridylic acid-agarose beads (Sigma-Aldrich). The assay involved incubation of 5 µg of each protein in 100 µL in a cold RNA-binding reaction buffer (10 mM HEPES, pH 7.4, 100 mM NaCl, 2 mM MgCl_2_, 0.1% Triton X-100, and 3 mM DTT). To evaluate the competition for binding sites, free poly(U) (Sigma-Aldrich) was added to the reactions at increasing concentrations of 0, 0.1, and 1 mg/mL and incubated for 20 min at 4 °C before the addition of the poly(U)-agarose beads and subsequent elution steps. Next, 10 µL of 50% poly(U)-agarose beads in RNA-binding buffer were added to each reaction mixture, followed by a 30-min incubation at 4 °C on a rotator. Afterward, the reaction mixtures were centrifuged at 9300 × g for 1 min at 4 °C, and the beads were then washed six times in RNA-binding reaction buffer, followed by centrifugation under the same conditions. Finally, the proteins bound to the poly(U)-agarose beads were eluted by adding 10 µL of 6 × SDS-PAGE sample buffer (60% glycerol, 12% SDS, 3% DTT, 1/8 v/v 0.5 M Tris, pH 6.8, bromophenol blue) and heated at 70 °C for 10 min. Subsequently, the samples were loaded onto a 12% SDS-PAGE gel and visualized using Coomassie brilliant blue staining. As a negative control, BSA was used, which did not demonstrate any binding activity, while the RNA binding protein HsaDRG1 served as a positive control.

### Cell culture and transfection procedure

MCF-7 (ECACC cat. no. 86012803) and MDA-MB-231 (ATCC cat. no. HTB-26) cell lines were grown in Dulbecco’s Modified Eagle Medium with high glucose (DMEM, Sigma-Aldrich) supplemented with 10% fetal bovine serum (FBS, Capricorn Scientific), 1% nonessential amino acids (Sigma-Aldrich), and 1% antibiotic/antimycotic solution (Capricorn Scientific) at 37 °C with 5% CO_2_ in the humidified atmosphere. For cell transfection, the cells were seeded into 6-well, 24-well, or 96-well plates in a growth medium. After 24 h, transfection was performed using Lipofectamine 2000 or Lipofectamine 3000 (Thermo Fisher Scientific) following the manufacturer's protocol. The transfected cells were incubated for an additional 24 or 48 h.

### Immunofluorescence and confocal microscopy

Immunofluorescence analysis of MCF-7 and MDA-MB-231 cells transfected with fluorescently labelled sponge (EsuRRAS2-like-GFP) or human R-RAS2 (HsaRRAS2-GFP), or co-transfected with EsuRRAS2-like-CHERRY and HsaRRAS2-GFP, was conducted following previously established protocols [31, 32]. The primary and secondary antibodies, and stains used are listed in Additional file 2: Table S4. Confocal images were acquired using the laser scanning confocal microscope Leica TCS SP8 (Leica Microsystems, Wetzlar, Germany). Interference reflection microscopy (IRM) images were taken to determine the location of cell adhesions. Further image processing was conducted using the ImageJ software (National Institutes of Health). Colocalization between human or sponge R-RAS2 and endosomal markers was quantified using Coloc2 plugin and shown as Pearson’s correlation coefficient.

### SDS-PAGE and Western blot analysis

For Western blot analysis, 5 × 10^5^ of MCF-7 cells were seeded into a 6-well plate and transfected. Twenty-four hours after transfection, cell lysates were prepared as already described [31, 32] and loaded onto freshly prepared 8% and 12% gels or 4–15% precast polyacrylamide gels (Bio-Rad), separated by SDS-PAGE and transferred to nitrocellulose (Bio-Rad) or PVDF membranes (Roche). To confirm equal loading of proteins, the membranes were stained with AmidoBlack (Sigma-Aldrich). Upon blocking in 0.2% (w/v) I-block (Thermo Fisher Scientific), the membranes were incubated with the appropriate primary antibodies, followed by secondary antibodies (Additional file 2: Table S4). Chemiluminescence signals were visualized using an ECL blotting substrate (GE Healthcare) and captured on a UVItec Cambridge documentation system. The intensity of protein signals was quantified using the ImageJ software (National Institutes of Health). All raw images of Western blot data are shown in Additional file 5.

### MTT assay

For cell proliferation analysis using MTT (3-(4,5-dimethylthiazol-2-yl)-2,5-diphenyltetrazolium bromide) (Millipore) assay, 4 × 10^3^ of MDA-MB-231 cells were seeded into a 96-well plate, transfected with EsuRRAS2-like-FLAG or HsaRRAS2-FLAG construct and further processed as already described [32].

### Colony formation assay upon R-RAS2 transfection

For the colony formation assay, MDA-MB-231 cells were initially seeded into a 6-well plate and transfected with EsuRRAS2-like-FLAG or HsaRRAS2-FLAG construct. Twenty-four hours after transfection, cells were resuspended and seeded into 60 mm Petri dishes at the density of 5 × 10^4^ cells per dish in a growth medium supplemented with 500 µg/mL G418 (Neomycin, Sigma-Aldrich) for the selection of resistant colonies. The cells were incubated for ten days, allowing the colonies to form. After the incubation, the resistant colonies were fixed with 100% methanol for 10 min, dried, stained with 10% Giemsa (Sigma-Aldrich) for 30 min, and then counted.

### Cell migration assays

For monitoring cell migration, wound healing and Boyden chamber assay were used. A wound healing assay was performed by seeding 5 × 10^4^ MDA-MB-231 cells into a 24-well plate which were than transfected with an EsuRRAS2-like-FLAG or HsaRRAS2-FLAG construct. Twenty-four hours after transfection, a wound was created by scratching the cell monolayer in a straight line using a sterile 100 μL pipette tip. The cells were washed three times with fresh medium and incubated under growth conditions for 24 h. After the 24-h incubation period, cell migration into the cell-free area was observed using 100 × magnification on the microscope (Olympus CKX41, Tokyo, Japan). The gap closure was measured between two margins of each scratch in five points after 24 h and compared with gaps in the same fields at the time point zero using the ImageJ software (National Institutes of Health).

For the Boyden chamber assay, MDA-MB-231 cells were seeded into a 6-well plate and transfected with the aforementioned constructs and starved overnight by replacing the growth medium with DMEM without FBS. Thirty-six hours after transfection, the cells were resuspended in DMEM and seeded into migration Transwell Cell Culture Inserts (pore size 8 mm, Corning) at the density of 2.5 × 10^4^ cells per well. Subsequently, the cells were left to migrate for 24 h toward a chemoattractant, DMEM supplied with 10% FBS. The cells that migrated to the underside of the filter were fixed with 4% sucrose/paraformaldehyde, stained with 1% crystal violet solution, and then subjected to imaging at 200 × magnification using the microscope (Olympus BX51, Tokyo, Japan). The extent of cell migration was quantified using the ImageJ software (National Institutes of Health).

### Isolation of focal adhesions

Focal adhesions were isolated from cells as previously described [33, 34]. Briefly, MDA-MB-231 cells were seeded (1.4 × 10^6^) in uncoated Petri dishes and upon 24 h transfected with empty pcDNA3.1 expression vector, or vector containing either FLAG-tagged sponge (EsuRRAS2-like-FLAG) or human R-RAS2 (HsaRRAS2-FLAG). Upon 24 h cells were washed with DMEM-HEPES and incubated with Wang and Richard’s reagent (DTBP, 6 mM, Thermo Fisher Scientific) for 10 min. DTBP was quenched with 0.03 M Tris–HCl (pH 8) and cells were lysed using modified RIPA buffer (50 mM Tris–HCl, pH 7.6; 150 mM NaCl; 5 mM disodium EDTA, pH 8; 1% (w/v) Triton X-100, 0.5% (w/v) SDS, 1% (w/v) sodium deoxycholate). Cell bodies were removed by high-pressure washing with tap water for 10 s and remaining adhesion complexes were collected by scraping into adhesion recovery solution (125 mM Tris–HCl, pH 6.8; 1% (w/v) SDS; 150 mM dithiothreitol). Samples containing isolated focal adhesions were acetone-precipitated and dissolved in 2 × sample buffer and further processed for SDS-PAGE and WB analysis [35].

### Statistical analysis

The statistical data analysis was conducted using the SPSS statistical package for Windows (v17.0). All biological experiments were carried out in 2 or 3 biological replicates and repeated threefold to ensure reliability. To compare the means of two independent groups and to determine if they differ from each other, the t-test was employed. The threshold for statistical significance was set at *p* < 0.05 for all analyses.

## Results

### The sponge R-RAS2-like protein, a single homolog of R-RAS subfamily members, is most closely related to the vertebrate R-RAS2 protein

**Fig. 1:**
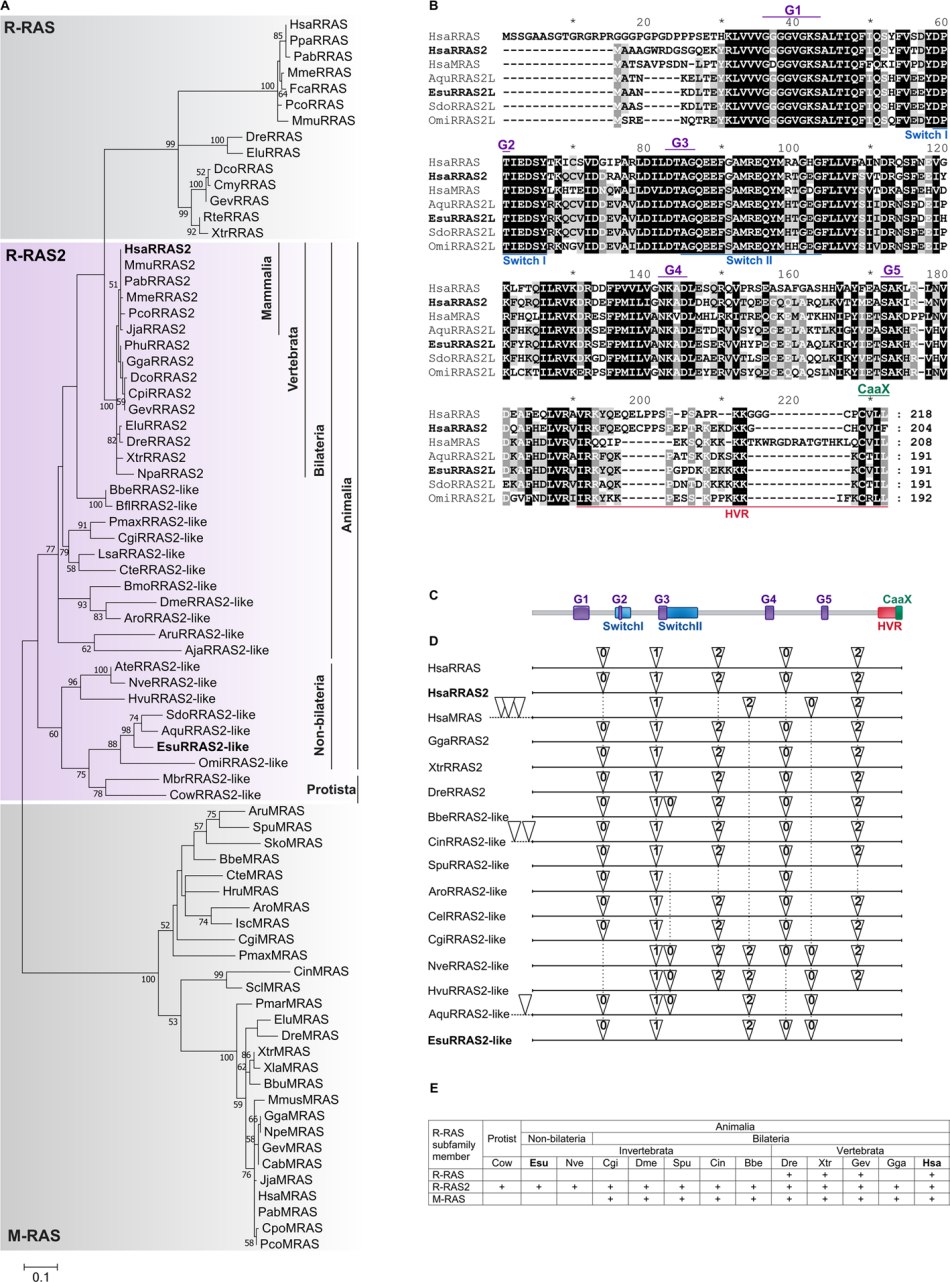
Evolutionary analysis of R-RAS subfamily proteins. **A** Phylogenetic relationships of R-RAS subfamily proteins in Metazoa and Protista. The representatives of R-RAS2-like and R-RAS2 proteins are highlighted in purple, with corresponding taxonomic groups indicated. The R-RAS and M-RAS proteins are shaded in grey. ML bootstrap values, based on 1000 bootstrapping replications, are represented as numbers associated with the branches (bootstrap values higher than 50% are displayed at the branching points). The scale bar denotes the number of substitutions per site. Additional file 2: Table S1 provides the accession numbers of the amino acid sequences used in our study. **B** Multiple sequence alignment of R-RAS2-like proteins from sponges and human R-RAS subfamily members. Conserved domains are indicated as follows: G motifs are shown in purple above the alignment, *Switch* regions in blue below the alignment, hypervariable region (HVR) in red below the alignment, and CaaX box in green above the alignment. **C** A schematic representation of human R-RAS2 protein with indicated conserved domains (above) and **D** the structure of *r-ras2* and *r-ras2-like* genes from selected metazoans (below). Triangles mark the positions of introns, and the number within each triangle represents the intron phase. Black dashed lines connect introns that share the same positions and phases based on the alignment of amino acid sequences. The sequences of genes with corresponding intron positions were obtained from the NCBI's genomic database. **E** A table showing the number of R-RAS subfamily members among different species

The mammalian Ras-related subfamily encompasses R-RAS, R-RAS2, and M-RAS proteins. To elucidate the evolution of these proteins, we performed a phylogenetic analysis using representatives of the R-RAS subfamily from selected metazoans, as well as from their closest unicellular relatives. Among non-bilaterian animals (phyla Porifera and Cnidaria), a single homolog of this subfamily was identified, likely representing the ancestral type protein of all three members present in the human genome. Based on our bioinformatics analysis (Fig. 1), this single homolog is most closely related to the human R-RAS2 protein. Therefore, we named it R-RAS2-like protein.

Our phylogenetic analysis indicated that M-RAS likely diverged from this common ancestral R-RAS2-like protein within Bilateria, based on the presence of M-RAS homologs in lineages from annelids to mammals (Fig. 1A, E). The M-RAS proteins formed a distinct and well-supported clade (bootstrap value 100%) separate from other R-RAS subfamily members, indicating their independent evolution within the R-RAS subfamily. Our analysis showed that R-RAS and R-RAS2 diverged from a common R-RAS2-like protein much later in evolution, during the transition to Osteichthyes, indicating their potential functional specialization in Vertebrata (Fig. 1A, E). This divergence allowed the acquirement of unique properties of R-RAS and R-RAS2, enabling them to participate in specific cellular processes. Interestingly, birds exhibit a notable absence of the R-RAS protein, which is present in the genomes of most other vertebrate lineages (Fig. 1A, E). Avian genomes are characterized by a reduction in protein-coding genes and possess fewer members in some other gene families [36, 37]. Many of these genes play critical roles in inducing lethality in rodents, human genetic disorders, or exhibit tissue-specific biological functions [38]. In our phylogenetic analysis, R-RAS2-like proteins from sponges formed a clade with homologs from protists closely related to animals. Additionally, these proteins formed a sister group with homologs from other non-bilaterian animals. Representatives of R-RAS2-like proteins from bilaterians were placed in a separate clade. A divergence within vertebrates resulted in the appearance of two already mentioned paralogs, R-RAS and R-RAS2. The branches representing these paralogs showed high bootstrap values (99% and 100%, respectively), which support the inferred relationships in the phylogenetic tree.

The R-RAS2-like protein identified in the sponge *E. subterraneus* comprises 191 amino acids. As sponges have only one R-RAS subfamily member, we conducted a comparative analysis to determine its similarity to the three corresponding members in the human genome. Sponge R-RAS2-like protein shares the highest homology (78.4%) with human R-RAS2, followed by 71.6% homology with M-RAS and 64.2% homology with R-RAS (Additional file 3). To further investigate the evolutionary conservation of regions involved in the GTP binding and hydrolysis, which are common among all RAS superfamily members, we compared homologs from sponges and humans. We found that G-motifs are highly conserved between sponges and human R-RAS subfamily members, with minor differences observed in the G1 and G4 motifs of the human M-RAS protein (Fig. 1B). This observation is consistent with our phylogenetic analysis, as M-RAS represents the most evolutionary distant member of the R-RAS subfamily. In addition, we found that the *SwitchI* region is highly conserved (100% identity across all analyzed sequences), while the *SwitchII* region is also conserved, although showing lower sequence identity. *Switch* regions are highly dynamic and change their conformation upon GTP binding and hydrolysis. Of particular note are the conserved *SwitchI* threonine (T46 in HsaRRAS2, corresponding to T35 in canonical Ras proteins) and *SwitchII* glycine (G71 in HsaRRAS2, corresponding to G60 in canonical Ras proteins) which form hydrogen bonds with the γ-phosphate and hold *SwitchI* and *SwitchII* regions in the active conformation, respectively. Upon GTP hydrolysis, the γ-phosphate is released and both *Switch* regions return to the flexible conformation in the GDP-bound state [5, 39]. Significant differences in protein length were observed among members of the R-RAS subfamily, particularly the elongated N-terminal region of human R-RAS and the hypervariable region (HVR) at the C-termini of the analyzed proteins. Notably, within the HVR, both human R-RAS and R-RAS2 exhibit a conserved proline-rich motif, commonly referred to as the R-RAS box, which is absent in human M-RAS and the R-RAS2-like homologs in sponges. Following this proline-rich motif is the CaaX box, a sequence present in all representatives of the R-RAS subfamily, known to be crucial for their localization to the plasma membrane [6]. We further aligned the R-RAS2 and R-RAS2-like homologs from metazoans and their closest unicellular relatives, and our results confirmed significant conservation of the five G-motifs and two switch regions that are crucial for GTPase activity (Additional file 2: Fig. S1). Moreover, we observed that the proline-rich motif is present only in vertebrate R-RAS2 representatives and absent in R-RAS2-like homologs from lower metazoans. This indicates that the proline-rich region likely emerged during the divergence of R-RAS and R-RAS2 paralogs from a common ancestral R-RAS2-like protein. We also identified that the CaaX box, which plays an important role in protein localization, is conserved among all R-RAS2 and R-RAS2-like proteins, emphasizing its functional significance. A heatmap displaying multiple sequence alignments revealed a high overall protein sequence homology among R-RAS2 and R-RAS2-like proteins (Additional file 2: Fig. S2). The identity/similarity scores exceeded 50%, indicating evolutionary conservation and the significant cellular role of this protein. As expected, the highest homology was observed among vertebrate R-RAS2 proteins, ranging from 75 to 100% (Additional file 4).

We conducted a comprehensive structural analysis of *r-ras2* genes from selected vertebrates, along with *r-ras2-like* homologs from lower metazoan lineages (Fig. 1C, D). Considering that *r-ras* and *m-ras* genes emerged through divergence from a common *r-ras2-like* gene during animal evolution, we included human *r-ras* and *m-ras* genes in our analysis. We compared the intron–exon composition to determine whether the conservation of gene structure aligns with the observed protein conservation. Our results revealed that the human *r-ras2* gene possesses all five introns that were originally present in the common ancestral *r-ras2-like* gene found in non-bilaterian animals. Notably, three of these introns are shared between sponges and humans, demonstrating a conserved intron composition across these phylogenetically distant species. We also observed identical positions and phases of all introns within human *r-ras* and *r-ras2* genes, suggesting a close evolutionary relationship and relatively recent divergence. Interestingly, the human *m-ras* gene shares two introns with other human paralogs, while the remaining two introns are exclusively shared with *r-ras2-like* genes from non-bilaterian animals and are absent in *r-ras2-like* genes from bilaterians (Fig. 1D). These findings support our previous results and reinforce the hypothesis that *m-ras* diverged from the *r-ras2-like* gene during the transition from non-bilaterians to bilaterians. Our results contribute to a deeper understanding of the evolutionary dynamics and the conservation of gene structure within the *r-ras* gene subfamily.

### The sponge R-RAS2-like and human R-RAS2 have similar biochemical properties

**Fig. 2:**
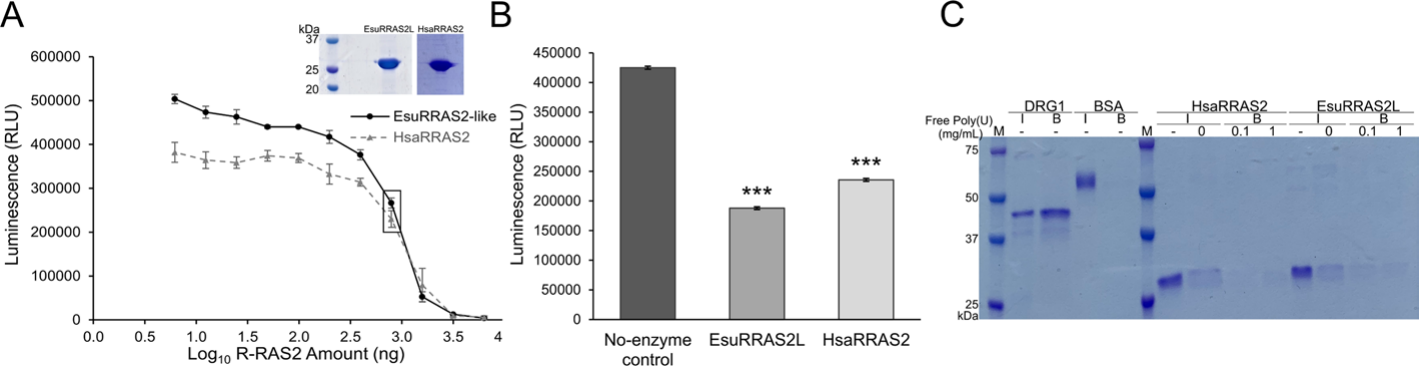
Biochemical properties of EsuRRAS2-like and HsaRRAS2 proteins. **A** Titration of EsuRRAS2-like and HsaRRAS2 proteins in GTPase/GAP Buffer with a molar concentration of 1 µM GTP. Successful isolation and purification of EsuRRAS2-like and HsaRRAS2 proteins confirmed by SDS-PAGE gel. Concentration of 6.25 µM (791.875 ng) is marked with rectangle. **B** Both the sponge and human R-RAS2 homologs exhibited intrinsic GTPase activity at a concentration of 6.25 µM. Luminescence was measured after a two-hour incubation. The control sample contained only the GTP/GAP buffer. Standard deviations are indicated as mean ± SD, *n* = 3. RLU (relative luminescence unit). ***p < 0.005. **C** RNA binding activity of sponge R-RAS2-like and human R-RAS2. Sponge and human proteins (5 µg) were preincubated with increasing concentrations of free poly(U), followed by incubation with 50% poly(U) agarose beads. The RNA binding protein DRG1 served as a positive control, while BSA served as a negative control. After incubation, proteins were analyzed by SDS-PAGE and stained with Coomassie Brilliant Blue. Abbreviations: I-input, B-beads

To analyze the biochemical properties of the EsuRRAS2-like protein and to compare it with human R-RAS2, we produced and purified both proteins (Fig. 2A). The GTPase activity of the sponge homolog was measured using the luminescence-based GTP hydrolysis assay. Initially, we titrated the HsaRRAS2 protein in the presence of 1 µM GTP to determine the optimal enzyme concentration for the reaction (Fig. 2A). Next, we analyzed the intrinsic GTPase activity of EsuRRAS2-like and HsaRRAS2 at concentrations of 6.25 µM. Using a luminescence-based GTP hydrolysis assay, we observed a significant reduction in luminescence signal for both EsuRRAS2-like and HsaRRAS2, indicating GTP hydrolysis (Fig. 2B). This demonstrated that ancient EsuRRAS2-like already possesses intrinsic GTPase activity, similar to its human homolog, and indicates a conserved role of GTPase activity of R-RAS2 in regulating similar signalling pathways and cellular processes in sponge and humans.

To evaluate the RNA-binding ability of sponge and human R-RAS2 proteins, we used polyuridylic acid (poly(U)) agarose beads, as described in previous studies [32, 40]. Our results show that both EsuRRAS2-like and HsaRRAS2 proteins exhibited binding affinity towards the poly(U) agarose beads, whereas BSA, which served as the negative control, did not. To further investigate whether RNA binding is specific, we introduced free poly(U) as a competitor during the binding assay. We observed a dose-dependent reduction in binding when R-RAS2 proteins were pre-incubated with increasing concentrations of free poly(U) before the addition of poly(U) beads (Fig. 2C). These results indicate that the interaction between R-RAS2 proteins and RNA is specific.

### The sponge R-RAS2-like and human R-RAS2 have similar but not identical localizations

**Fig. 3:**
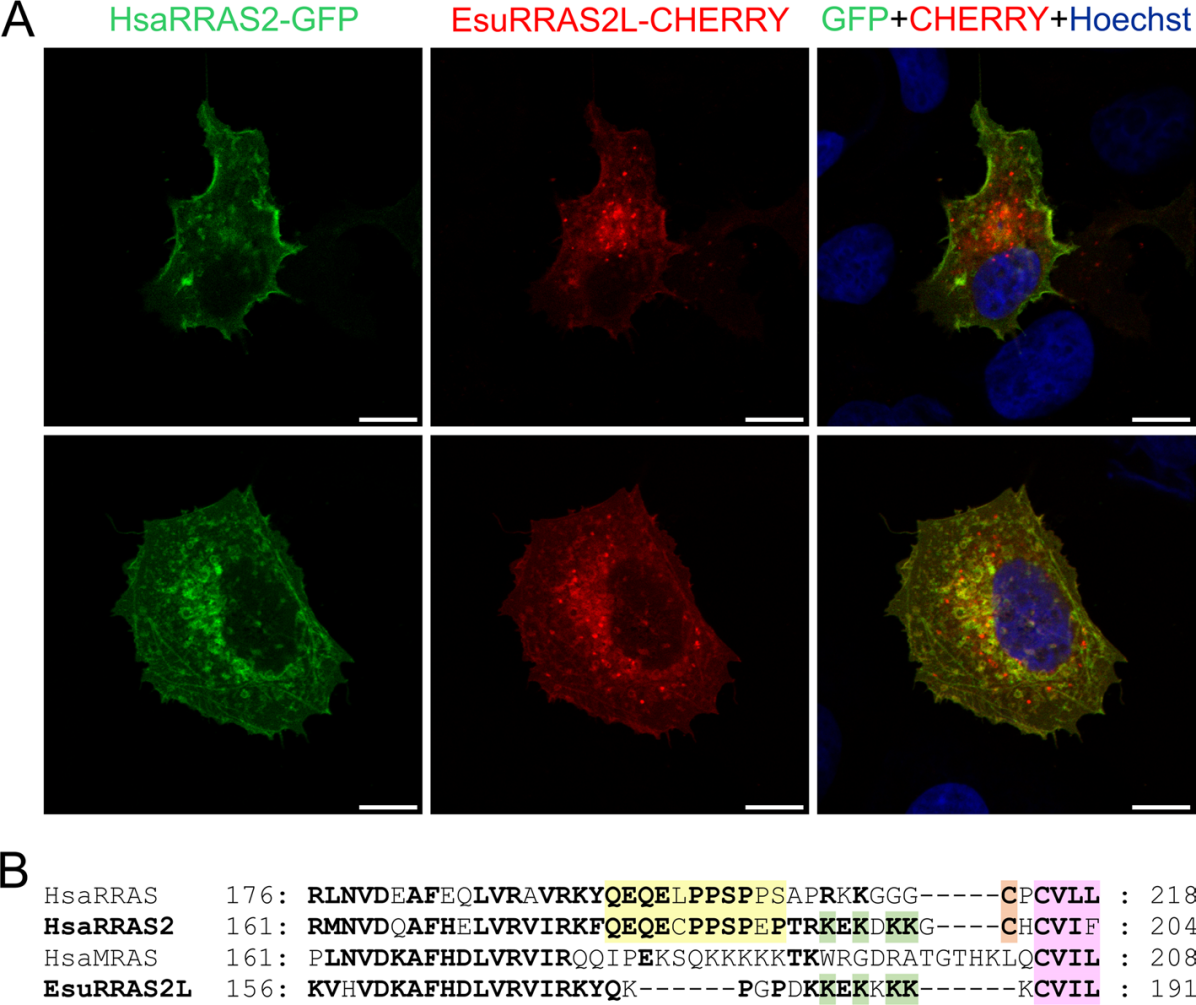
Sponge and human R-RAS2 homologs have similar but not identical localization in membranes of MCF-7 cells. **A** Colocalization (yellow) of human R-RAS2 with sponge homolog EsuRRAS2L in the membranes of human breast cancer cells MCF-7. Human R-RAS2 was fluorescently labelled with GFP, and sponge EsuRRAS2L was fluorescently labelled with CHERRY (red). Hoechst was used to stain nuclei. The experiments were repeated three times in biological duplicates. Cells were analyzed by confocal microscopy. **B** The C-terminal hypervariable region of R-RAS subfamily members. The proline-rich region, present in HsaRRAS and HsaRRAS2 proteins, is shown in yellow, while the cysteine residue that undergoes palmitoylation is shown in orange. Lysine residues conserved between sponge R-RAS2-like and human R-RAS2 are shown in green. The CaaX box conserved between all analyzed sequences is highlighted in pink. Esu-sponge *Eunapius subterraneus*, Hsa-human. Scale bar—10 μm

To determine the R-RAS2 protein localization in human tumor cells, we co-transfected MCF-7 and HeLa cells with EsuRRAS2-like fluorescently labelled with CHERRY, and HsaRRAS2 fluorescently labelled with GFP. We observed that the exogenous sponge and human homologs localize in the cytosol of MCF-7 and HeLa cells and not in the nucleus. The punctuate staining patterns indicate the association of EsuRRAS2-like and HsaRRAS2 with the plasma membrane and other intracellular membranes, likely the endocytic vesicles. Based on the staining morphology, the sponge homolog is more localized in intracellular membranes, whereas the human homolog is more localized in the plasma membrane (Fig. 3A and Additional file 2: Fig. S3). Therefore, we confirmed partial colocalization of EsuRRAS2-like and HsaRRAS2 in the membranes of MCF-7 and HeLa cells. The localization of EsuRRAS2-like mainly at endosomal membranes rather than at the plasma membrane may be due to two differences between these proteins. The first is the lack of a cysteine residue crucial for palmitoylation, while the second is a shorter HVR in the sponge EsuRRAS2-like protein (Fig. 3B), as already shown for human R-RAS [6].

**Fig. 4:**
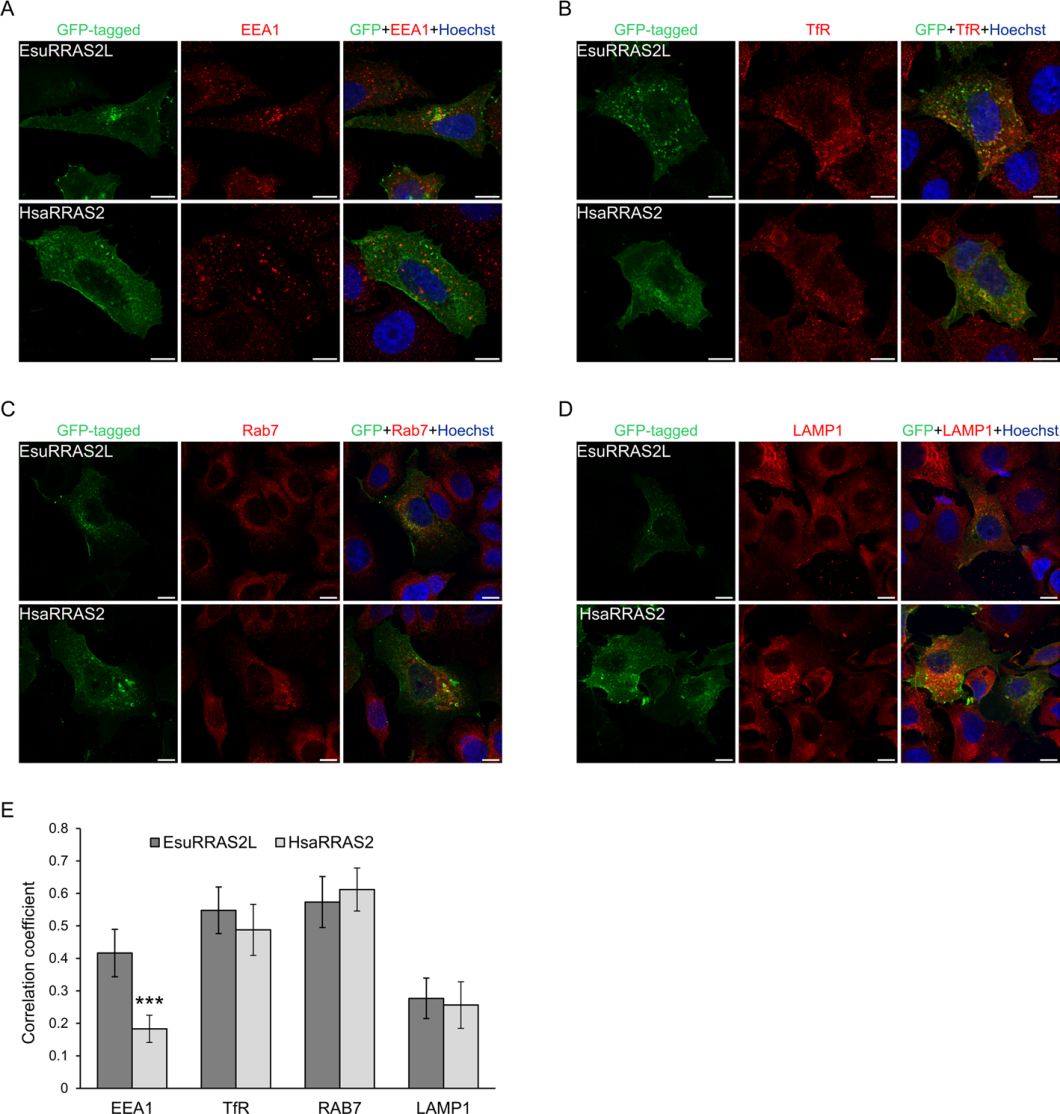
Both sponge and human R-RAS2 are localized within recycling and late endosomes. Colocalization (yellow) of sponge EsuRRAS2L or human HsaRRAS2 fluorescently labelled with GFP (green) with markers (red) for **A** early endosomes (EEA1), **B** recycling endosomes (TfR), **C** late endosomes (Rab7), and **D** lysosomes (LAMP1) in human breast cancer cells MCF-7. Hoechst was used to stain nuclei. **E** Quantification of colocalization between EsuRRAS2L or HsaRRAS2 with endosomal markers was done using ImageJ Coloc2 plugin and shown as Pearson’s correlation coefficient. ***p < 0.001, *n* = 30 cells per group from three different experiments. Cells were analyzed by confocal microscopy. Esu-sponge *Eunapius subterraneus*, Hsa-human. Scale bar—10 μm

We further analyzed the specific membranes in which EsuRRAS2-like and HsaRRAS2 proteins are localized. For that purpose, we co-stained MCF-7 cells transfected with GFP-labelled sponge or human R-RAS2 homologs with markers for endocytic vesicles (Fig. 4). We observed partial colocalization of EsuRRAS2-like with early endosomes (EEA1), which was not observed for HsaRRAS2 (p*** < 0.001, Fig. 4A, E). Our results demonstrated that both EsuRRAS2-like and HsaRRAS2 proteins exhibited the highest colocalization with recycling (TfR) and late endosomes (Rab7) (Fig. 4B, C, E) and no colocalization with lysosomes (LAMP1, Fig. 4D, E). The localization within recycling endosomes was confirmed using additional marker, RAB11 (Additional file 2: Fig. S4A, B). These results provide valuable insight into the precise subcellular localization of EsuRRAS2-like and HsaRRAS2 within the endosomal pathway.

**Fig. 5:**
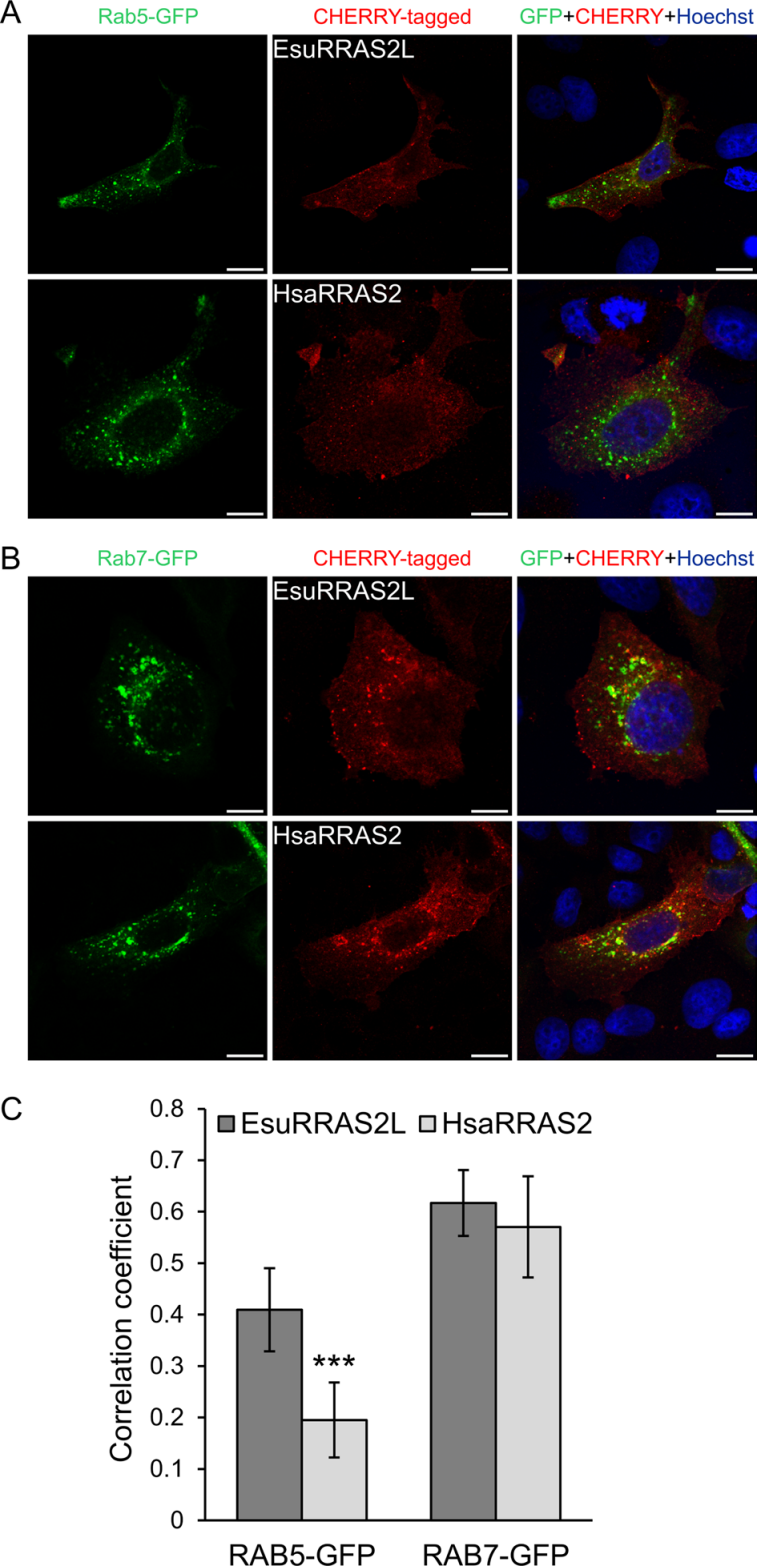
Both sponge and human R-RAS2 are localized within overexpressed early and late endosomal marker proteins. Colocalization (yellow) of cotransfected sponge EsuRRAS2L or human HsaRRAS2 fluorescently labelled with CHERRY (red) together with markers (green) for **A** early endosomes (Rab5), and **B** late endosomes (Rab7) in human breast cancer MCF-7 cells. Hoechst was used to stain nuclei. **C** Quantification of colocalization between EsuRRAS2L or HsaRRAS2 with endosomal markers was done using ImageJ Coloc2 plugin and shown as Pearson’s correlation coefficient. ***p < 0.001, *n* = 30 cells per group from three different experiments. Cells were analyzed by confocal microscopy. Esu-sponge *Eunapius subterraneus*, Hsa-human. Scale bar—10 μm

In order to distinguish whether these proteins participate in early or late endocytosis, we further wanted to analyze the localization of both EsuRRAS2-like and HsaRRAS2 within vesicles in MCF-7 cells. For this purpose, we co-transfected CHERRY-tagged EsuRRAS2-like or HsaRRAS2, along with GFP-tagged Rab5 (an early endosome marker, Fig. 5A) or Rab7 (a late endosome marker, Fig. 5B). Although we observed only partial colocalization within early endosomes, EsuRRAS2L shows statistically more colocalization with Rab5 than HsaRRAS2 (p*** < 0.001, Fig. 5A, C), our results confirmed the localization of EsuRRAS2-like and HsaRRAS2 within late endosomes (Fig. 5B, C). These results are in accordance with endosomal transport of proteins localized in both plasma and endosomal membranes [41].

**Fig. 6:**
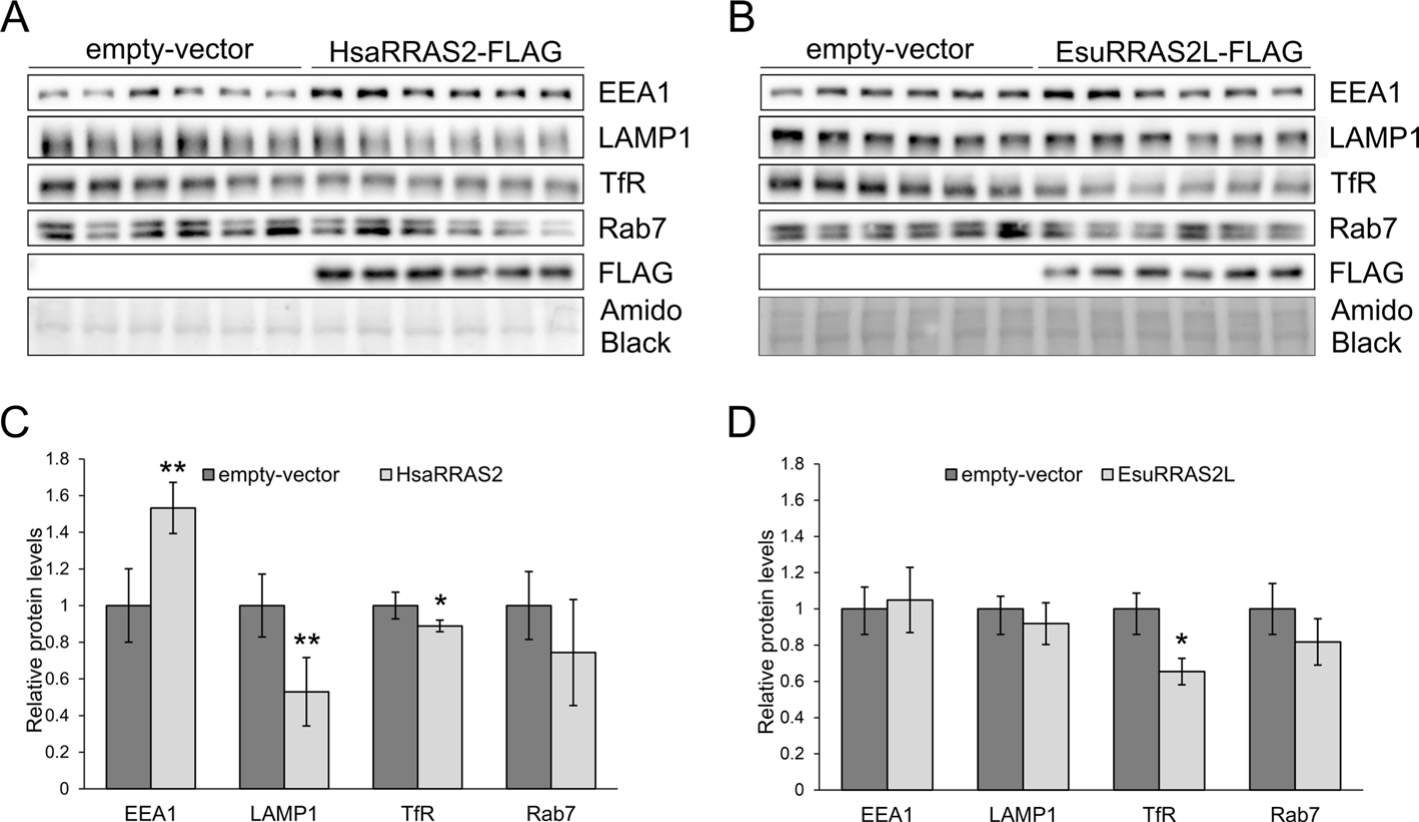
The expression of human and sponge R-RAS2 alters the expression of endosomal vesicles markers. Levels of overexpressed **A** human or **B** sponge R-RAS2 homologs labelled with FLAG, and endogenous levels of markers for endosomal vesicles: early endosomes (EEA1), lysosomes (LAMP1), recycling endosomes (TfR) and late endosomes (Rab7) were analyzed by Western blot and detected with specific primary antibodies. **C** and **D** Quantification for each protein levels was shown as ratio to empty-vector, *p < 0.05, **p < 0.01, ***p < 0.001. Amido Black was used as a loading control. Cropped blots are displayed. Esu-sponge *Eunapius subterraneus*, Hsa-human

To investigate the potential impact of EsuRRAS2-like and HsaRRAS2 overexpression on the expression levels of proteins associated with early, recycling, and late endosomes, and lysosomes (EEA1, TfR, Rab 11, Rab7, and LAMP1), we transfected MCF-7 cells with FLAG-tagged EsuRRAS2-like or HsaRRAS2 constructs and analyzed proteins of interest by Western blot analysis. We found that overexpression of HsaRRAS2 significantly reduced the levels of TfR (a marker for recycling endosomes, *p* < 0.001, Fig. 6A, C) compared to cells transfected with an empty vector. The overexpression of EsuRRAS2-like had a similar effect of reducing the levels of TfR (*p* = 0.0158) (Fig. 6B, D). However, neither HsaRRAS2 nor EsuRRAS2L change the expression levels of Rab11, another marker of recycling endosomes (Additional file 2: Fig. S4C, D, E, F). Furthermore, the overexpression of HsaRRAS2 caused increased protein levels of EEA1 (*p* = 0.0011) and decreased levels of LAMP1 (*p* = 0.0032) (Fig. 6A, C) while the sponge homolog EsuRRAS2-like did not (Fig. 6B, D). Therefore, human homolog impacts TfR level more than the sponge homolog, and the sponge homolog does not affect EEA1 and LAMP1 levels. Although our study shows that both exogenous HsaRRAS2 and EsuRRAS2-like influence the dynamics and turnover of endosomal vesicles in MCF-7 cells, further studies are necessary to clarify these observations.

### Main biological functions of the sponge R-RAS2-like and human R-RAS2 are conserved

**Fig. 7:**
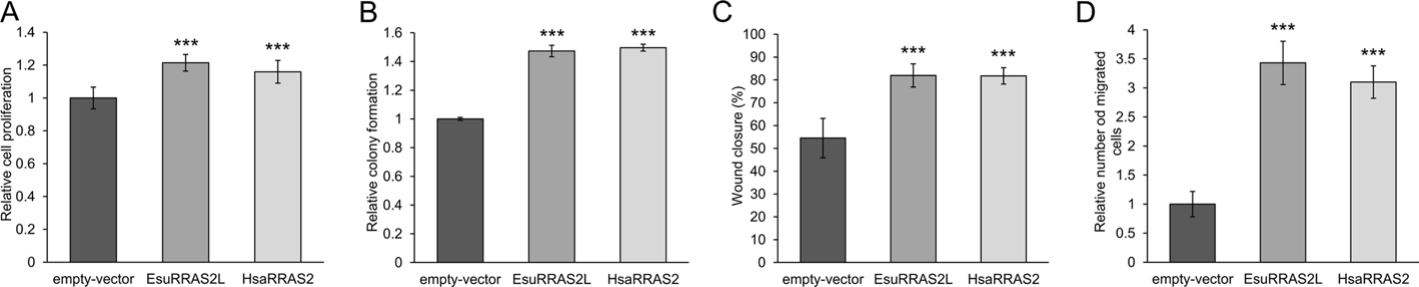
Both sponge and human R-RAS2 homologs display oncogenic properties in MDA-MB-231 cells. **A** cell proliferation, **B** number of colonies formed, **C** wound healing, and **D** cell migration was quantified using the ImageJ software (National Institutes of Health, USA). The statistical significance of the tests was set at *p < 0.05, **p < 0.01, ***p < 0.001. The experiments were repeated three times in biological duplicates. Esu-sponge *Eunapius subterraneus*, Hsa-human

Next, we investigated the biological effects of EsuRRAS2-like on cancer cells. For this purpose, we used MDA-MB-231 cells, the most commonly used triple-negative breast cancer cell line model, which carry mutations in Ras effector pathway (*BRAF* and *NF1* mutations). First, we analyzed the proliferation of cells transfected with FLAG-tagged EsuRRAS2-like and HsaRRAS2 constructs. A significant increase in cell proliferation was observed for both EsuRRAS2-like and HsaRRAS2 (*p* < 0.001) compared to an empty-vector (Fig. 7A). Next, we examined the impact of EsuRRAS2-like or HsaRRAS2 transfection on cell survival and colony formation. Similar to the cell proliferation results, both EsuRRAS2-like and HsaRRAS2 overexpression increased the number of colonies formed (*p* < 0.001, respectively) (Fig. 7B). To assess the role of EsuRRAS2-like and HsaRRAS2 in cell migration, we employed wound healing and Boyden chamber assays. MDA-MB-231 cells transfected with both EsuRRAS2-like and HsaRRAS2 exhibited enhanced cell migration and faster wound closure compared to control (*p* < 0.001) (Fig. 7C, D). These results confirm the conserved function of human R-RAS2 and its sponge homolog in tumor-related processes, indicating their potential oncogenic role. This reiterates the functional significance of R-RAS2 in cancer cells and highlight the conservation of its biological functions across animals.

### The sponge R-RAS2-like and human R-RAS2 regulate focal adhesions

**Fig. 8:**
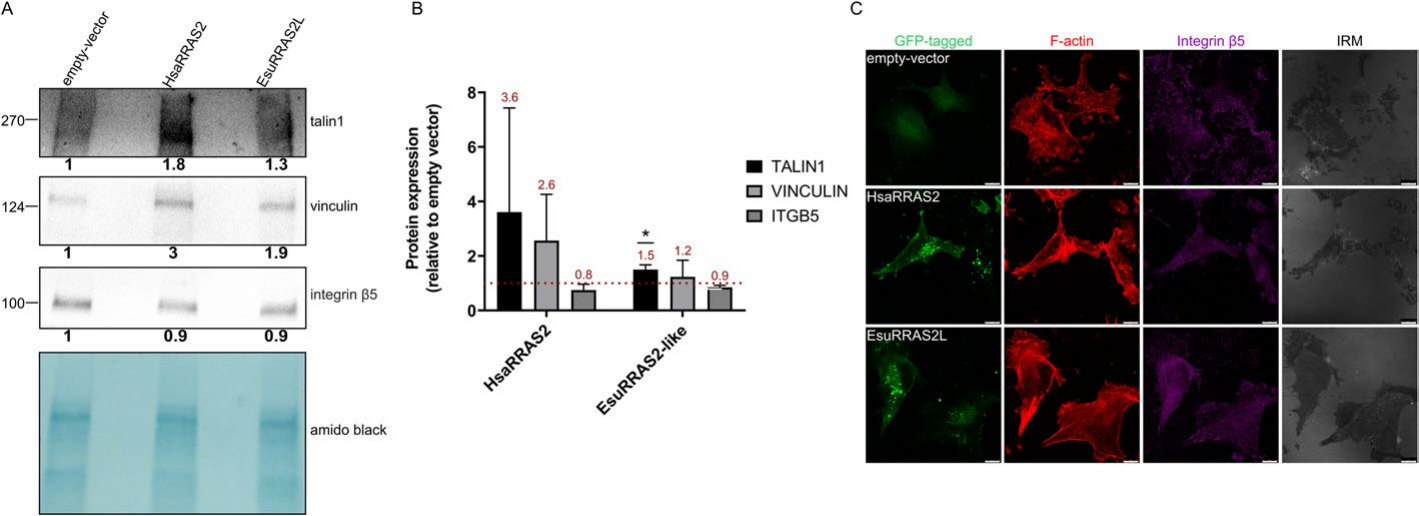
Both sponge and human R-RAS2 homologs regulate focal adhesions in MDA-MB-231 cells. **A** Twenty-four hours upon transfection with either empty vector of vector containing EsuRRAS2-like-FLAG or HsaRRAS2-FLAG construct, focal adhesions were isolated and WB analysis of talin1, vinculin and integrin β5 was performed. Numbers below blots represent the relative expression of proteins compared to control (empty-vector) normalized against amidoblack staining of focal adhesion isolates. Densitometry was done using the ImageJ software (National Institutes of Health, USA). **B** Quantification of Western blot data presented in (A) together with two independently performed biological replicas. Histogram data are plotted as mean ± SD (*n* = 3) relative to expression in cells transfected with an empty-vector that was set as 1 (indicated by a dotted line). Data were analyzed by unpaired Student’s t-test. *p < 0.05. **C** Forty-eight hours after transfection with either an empty-vector or a vector containing EsuRRAS2-like-GFP or HsaRRAS2-GFP construct, cells were fixed with paraformaldehyde and incubated with Alexa-Flour 488 conjugated phalloidin for F-actin visualization, anti-integrin β5 antibody followed by Alexa-Fluor 647-conjugated antibody and IRM images were taken. Analysis was performed using TCS SP8 Leica. Scale bar—10 μm. Esu-sponge *Eunapius subterraneus*, Hsa-human

It has recently been shown that endogenous R-RAS2 (upon R-RAS2 knock-out and knock-in of fluorescently labelled R-RAS2), unlike canonical Ras proteins, specifically localizes in focal adhesions [12]. However, in our experiments we did not observe that the sponge or the human homolog localizes in focal adhesions. In contrast, our data showed the association of EsuRRAS2-like and HsaRRAS2 with the plasma membrane and endocytic vesicles. More specifically, both EsuRRAS2-like and HsaRRAS2 proteins showed the highest colocalization with recycling (TfR) and late endosomes (Rab7) (Figs. 4B, C, E and 5B, C). To demonstrate the ability of transfected HsaRRAS2 or EsuRRAS2-like to regulate focal adhesions, we performed biochemical isolation of focal adhesions in MDA-MB-231 cells transfected with HsaRRAS2-FLAG or EsuRRAS2-like-FLAG constructs. The method is based on the use of the crosslinker DTBP, which diffuses into cells and crosslinks preferentially adhesion proteins. Cells are then lysed, washed with high pressure tap water to remove cell bodies, and focal adhesions are collected by scraping from Petri dishes [33]. All experiments were performed without prior coating of the growing surface with extracellular matrix proteins, as described in [34]. The samples were analyzed by Western blot using antibodies specific for two classic focal adhesion proteins, talin1 [42] and vinculin [43] as well as anti-FLAG antibodies. The overexpression of HsaRRAS2 in MDA-MB-231 cells significantly increased the level of talin1 and vinculin within focal adhesion isolates compared to cells transfected with the empty vector. A similar effect, although not as strong, was also observed after expression of EsuRRAS2-like (Fig. 8A). This result is consistent with the effect of transfected EsuRRAS2-like or HsaRRAS2 on cell proliferation, migration and colony formation (Fig. 7). MDA-MB-231 cells preferentially use integrin αVβ5 for adhesion [44] and for this reason we analyzed integrin subunit β5 expression within focal adhesion isolates. Surprisingly, the expression levels of integrin β5 did not change (Fig. 8A). Results of Western blot analysis obtained from pooled data from three independent experiments confirmed these observations (Fig. 8B). Due to the nature of the experiment involving transfection, lysis, cross-linking, washing and Western blot, standard deviations are large which affects statistical analysis. We were unable to demonstrate EsuRRAS2-like-FLAG or HsaRRAS2-FLAG expression in focal adhesion isolates, using anti-FLAG antibodies, indicating that EsuRRAS2-like and HsaRRAS2 do not localise within but regulate the formation of focal adhesions, however, not those formed by integrin heterodimers αVβ5.

To further investigate whether transfected R-RAS2 homologs regulate focal adhesions, we transfected MDA-MB-231 cells with HsaRRAS2 or EsuRRAS2-like fluorescently labelled with GFP and visualized the F-actin cytoskeleton and integrin αVβ5 focal adhesions. At the same time, IRM was used to additionally visualize focal adhesions (Fig. 8C). Again, we did not find the fluorescently labelled R-RAS2 homologs in focal adhesions. However, due to the expression of HsaRRAS2 or EsuRRAS2-like, we observed slightly altered appearance of actin stress fibers compared to cells transfected with a control plasmid. In our hands, the quantification of integrin αVβ5 focal adhesions or actin stress fibers in MDA-MB-231 cells was not possible. However, a melanoma cell line MDA-MB-435S also preferentially uses integrin αVβ5 for adhesion [34] and contains a very large number of focal adhesions with highly pronounced stress fibers. Therefore, to further analyze whether any of R-RAS2 homologs localize in and/or regulate focal adhesions, we visualized transfected HsaRRAS2 or EsuRRAS2-like fluorescently labelled with GFP together with actin stress fibers. The IRM and also integrin β5 were used to visualize focal adhesions. Similarly, as we observed in MDA-MB-231 cells, in MDA-MB-435S cells we could not find the fluorescently labelled R-RAS2 homologs in focal adhesions, but similarly we observed slight changes in organisation of F-actin (Additional file 2: Fig. S5A). However, quantification of integrin αVβ5 focal adhesions and actin stress fibers in MDA-MB-435S cells transfected with HsaRRAS2 or EsuRRAS2L showed that the number and size of integrin αVβ5 focal adhesions did not change, nor did the total amount of actin stress fibers (Additional file 2: Fig. S5B). Therefore, the sponge and human R-RAS2 homologs, HsaRRAS2 and EsuRRAS2-like, do not localise within focal adhesions but play a role in their regulation, but not those formed by integrin heterodimer αVβ5. It remains to be investigated which focal adhesions are altered and the sequence of events that leads to increased migration after overexpression of either HsaRRAS2 or EsuRRAS2L.

## Discussion

The objective of this study was primarily to determine the structure, biochemical, and biological characteristics of the ancestral type of the R-RAS protein, R-RAS2-like from the sponge *Eunapius subterraneus*, and compare it to the human homolog. The results should facilitate a deeper insight into the evolution of metazoan R-RAS proteins and their functions in cancer. Sponges, one of the most ancient and simplest animal groups, provide valuable insights into the origin and early evolution of multicellularity in the animal lineage. Despite lacking true tissues, organs, germ layers, and recognizable nerve structures, sponges possess complex genomes that contain numerous genes highly similar to their vertebrate homologs, including those implicated in early tumor development and progression [45–48]. Our previous studies have revealed that several sponge proteins show significant similarities in biochemical and biological characteristics when compared to their human homologs. These shared features strongly suggest that certain sponge proteins possess functions associated with metastasis or tumor suppression in human [32, 49–51].

Our analysis of the evolutionary history of the R-RAS subfamily members has shown that duplications and diversifications of the ancestral type of the R-RAS2-like protein to R-RAS, R-RAS2, and M-RAS occurred twice in metazoans. M-RAS arose by duplication of the R-RAS2-like protein during the transition to Bilateria, while the more recent R-RAS and R-RAS2 arose by duplication of the R-RAS2-like during the transition to Vertebrata. Our intron/exon structure analysis revealed that five introns found in the *r-ras2* genes of metazoans are ancient, as they were also present in non-bilaterian homologs, suggesting that the ancestral metazoan *r-ras2* gene was intron-rich.

Our biochemical characterization of sponge and human R-RAS2 shows their identical properties. The sponge R-RAS2-like has the same intrinsic GTPase activity as the human homolog. Che and coworkers [52] showed that K-Ras, a representative of the canonical Ras proteins, localizes from recycling endosomes to the plasma membrane through direct interactions with specific SNARE (Soluble N-ethylmaleimide attachment protein receptor) proteins. They also found that specific small non-coding RNAs can affect the subcellular trafficking of K-Ras by competing with SNARE proteins to bind K-Ras, and thus control the K-Ras-driven tumorigenesis. This is the first evidence of RNA binding capacity to the Ras superfamily of GTPases. Therefore, to further explore the RNA-binding ability of Ras GTPases, we investigated whether the sponge and human R-RAS2 can bind RNA homopolymer. Although R-RAS2 proteins lack the characteristic domains commonly found in RNA/DNA binding proteins, our results showed their ability to interact with RNA, suggesting the possible involvement of different mechanisms in the RNA-binding process. Further investigations are needed to elucidate the precise molecular interactions underlying R-RAS2 RNA-binding ability and explore its potential functional implications in cellular processes.

To test the biological function of the ancestral type of R-RAS2 protein and compare it to human R-RAS2, we analyzed function of exogenous sponge R-RAS2-like compared to human R-RAS2 in human tumor cell lines. We have found differences in their membrane distribution, as sponge R-RAS2 localized more in endosomal vesicles, while human R-RAS2 localized more in the plasma membrane. This difference can be explained by posttranslational modifications. All Ras proteins contain the carboxy-terminal CaaX motif that is modified by prenylation (farnesylation or geranylgeranylation) at the cysteine residue. Some Ras proteins undergo cysteine palmitoylation as a secondary lipidation. These two modifications play a crucial role in directing Ras proteins to the plasma membrane. However, certain Ras family proteins lack cysteine palmitoylation, instead, they are believed to use a C-terminal polybasic sequence for targeting the membrane. Interestingly, in addition to palmitoylation on Cys199 and farnesylation on Cys201, R-RAS2 also contains several lysine residues at the C-terminus (Lys192, 194, 196, 197) that may enable membrane targeting of R-RAS2 through lysine fatty acylation [3, 10]. Our analysis of the C-terminal HVR of R-RAS subfamily proteins confirmed that the ancestral type EsuRRAS2-like protein possesses a conserved CaaX motif, important for prenylation and membrane targeting (Fig. 3B). However, unlike the human R-RAS and R-RAS2 proteins, this ancestral protein lacks a cysteine residue upstream of the CaaX motif, which undergoes palmitoylation as a secondary modification for membrane targeting. However, we confirmed that unique lysine residues at the C-terminus of R-RAS2 (Lys192, 194, 196, 197), which are absent in R-RAS and M-RAS, were also found in the sponge R-RAS2-like homolog, indicating their probable involvement in facilitating proper localization. Furuhjelm and Peranen [6] highlighted the importance of cysteine palmitoylation and the hypervariable region (HVR) for the transport of R-RAS from endomembranes to the plasma membrane (from Golgi onwards). Since EsuRRAS2-like lacks the cysteine residue crucial for palmitoylation and has a shorter HVR, this could explain its accumulation and localization mainly at endosomal membranes rather than at plasma membrane. To validate this hypothesis and enhance our understanding of the R-RAS2 subcellular localization, additional experiments are required.

In addition to oncogenic mutations, overexpression of the wild-type R-RAS2 induces breast cancer transformation [53]. Therefore, we studied oncogenic potential of both human and sponge R-RAS2 homologs in breast cancer cell line MDA-MB-231. We observed that both proteins increase cell proliferation, number of colonies formed, wound healing and cell migration to a similar extent. Although there are just a few studies on the molecular mechanisms of R-RAS2 function in human cancer cells, our results are in accordance with the previously shown increase in cell motility of hepatocellular carcinoma cells [54], migration of *Nf1*-null Schwann cells [55], and migration and invasion of breast epithelial cells [56], due to overexpression of wild-type R-RAS2. Most of the studies focus on the expression levels of R-RAS2 in cancer tissues. Elevated levels of wild-type R-RAS2 were observed in various cancers, including esophageal tumors [21], oral cancer [22], skin cancer [23], lymphoma [24], and chronic lymphocytic leukemia (CLL) [20]. However, our study using sponge homolog shows that the ancestral type of the R-RAS protein, R-RAS2-like, has the same functions as human R-RAS2, suggesting that the function of R-RAS2 in oncogenic processes was present before the emergence of true tissues. Therefore, our results highlight the importance of additional studies into molecular mechanisms by which R-RAS2 can influence cell growth, proliferation and migration, or immunological development and homeostasis [20], via the PI3K signaling pathway, or beyond.

The hypervariable region of R-RAS plays a crucial role in both focal adhesion targeting and integrin activation [6]. Unlike most other members of the Ras superfamily, R-RAS has a characteristic proline-rich motif within its HVR. This proline-rich motif contains an SH3-binding site essential for R-RAS integrin activation [57]. This motif is also present in the human R-RAS2 protein (Figs. 1B and 3B). Furthermore, in contrast to previous reports using ectopic expression systems that showed subcellular localization of R-RAS2 protein in the plasma membrane, Golgi apparatus, or endoplasmic reticulum [9, 10], a recent study showed that endogenous wild-type R-RAS2 protein is specifically localized in both nascent and mature focal adhesions. The distribution of R-RAS2 overlaps with the focal adhesion marker vinculin and with F-actin present in these structures and regulates focal adhesion- and invasion-related functions [12]. This is in line with previous detection of R-RAS2 in proteomics experiments in myosin II-responsive focal adhesion complexes [58].

In light of these new data and the fact that a proline-rich motif is also present in the human R-RAS2 protein but absent from the sponge R-RAS2-like homolog (Figs. 1B and 3B), the finding that neither R-RAS2 homolog is localized in focal adhesions, was unexpected. However, Clavaín et al. [12] localized endogenous R-RAS2 proteins, wild type and R-Ras2Q72L mutant in focal adhesions in immunofluorescent analysis after seeding cells to fibronectin, which is a receptor for α5β1 [59] but not for αVβ5 integrin [60]. Since both cells used in our experiments, MDA-MB-231 and MDA-MB-435S, when grown on plastic use preferentially integrin heterodimer αVβ5 for adhesion [34, 44], this may be the explanation why we did not observe the localization of R-RAS2 proteins in focal adhesions.

We showed that overexpressed HsaRRAS2 and EsuRRAS2-like play a role in focal adhesion regulation. Using an improved approach to that performed by [58] for the isolation of focal adhesions using a crosslinking agent [33], we showed that transfection of MDA-MB-231 cells with HsaRRAS2 or EsuRRAS2-like enhance the amount of focal adhesion marker vinculin [43], as well as the amount of talin1, which is known to be crucial for integrin activation [42]. However, the amount of integrin β5 did not change, indicating that alterations in focal adhesions are not connected to integrin αVβ5. This conclusion is further supported by the results in melanoma cell line MDA-MB-435S. The transfection of these cells with HsaRRAS2 or EsuRRAS2-like did not alter neither number or size of integrin αVβ5 focal adhesions. Interestingly, both sponge and human R-RAS2 homologs seems to induce slight alteration of stress fibers appearance (F-actin), but quantification of actin stress fibers in MDA-MB435S cells did not support this observation. It is likely that simple quantification from immunofluorescent images is not an appropriate method to observe changes induced in transiently overexpressed R-RAS2. Therefore, these results of increased amount of two focal adhesion proteins, talin1 and vinculin, should be taken only as indication of alteration in focal adhesions that should be investigated in the future. However, functional tests of increased proliferation and migration after HsaRRAS2 or EsuRRAS2-like overexpression support these data. It is interesting that Miller and coworkers [61] showed that sponge homolog of vinculin interacts with talin and actin in cell-ECM adhesions. Therefore, our findings in MDA-MB-231 cells are in line with oncogenic potential of R-RAS2 homologs through regulation of focal adhesions. Talin1 was analyzed because it has been shown to be crucial in tumor cell adhesion and metastasis. More specifically, TLN1 knockdown significantly inhibited proliferation, cell adhesion and metastasis in vivo and in vitro in MDA-MB-231 cell model*.* Moreover, in TNBC patients, talin1 overexpression is associated with malignant behavior, including proliferation, cell adhesion, epithelial-to-mesenchymal transformation, invasion, migration and poor survival [62].

Given that R-RAS2 homologs are not found within focal adhesions, the mechanism by which they regulate focal adhesions remains to be determined. The likely mechanism is through the regulation of endocytosis. Focal adhesions are highly dynamic and undergo constant cycles of assembly and disassembly, referred as recycling. Adhesion turnover is in part regulated by endocytosis and exocytosis of integrins [63] which might be affected by R-RAS2 expression.

## Conclusions

By applying bioinformatics, biochemical and biological methods in this study, we can conclude four major points: (i) the single sponge R-RAS2-like protein most likely reflects the characteristics of the ancestral R-RAS protein, from which all three R-Ras subfamily members in the human genome arose; (ii) the sponge R-RAS2-like protein shows localization mainly at endosomal membranes rather than at plasma membrane; (iii) the sponge R-RAS2-like enhanced the oncogenic potential of MDA-MB-231 cells, same as human R-RAS2, suggesting that the function of R-RAS2 in oncogenic processes was engaged long before the composition of true tissues.; (iv) the sponge and human R-RAS2 homologs play a role in the regulation of focal adhesions contributing to metastasis and worse prognosis. This study suggests that the ancestor of all animals possessed an R-RAS2-like protein with similar properties and functions to the evolutionarily most recent versions of the protein, even before the appearance of true tissues and the origin of tumors.

### Supplementary Information

**Additional file 1. **Accession numbers and amino acid sequences of R-RAS proteins from selected species.

**Additional file 2: Figure S1.** Multiple sequence analysis of R-RAS2 and R-RAS2-like homologs from selected species. The conserved regions are indicated above the alignment, starting with the N-terminus: G1 motif, *SwitchI* region, G2 motif, G3 motif, *SwitchII* region, G4 motif, G5 motif, hypervariable region (HVR), CaaX box. The R-RAS2 and R-RAS2-like amino acid sequences were aligned using the ClustalX 2.0, and the alignment was visualised using the GeneDoc v2.7. Similar amino acids conserved among the analyzed proteins at levels of 100%, 80%, and 60% are represented as white letters on black background, white letters on dark grey background, and black letters on light grey background, respectively. **Figure S2.** Heatmap of amino acid sequence similarity and identity for R-RAS2-like and R-RAS2 proteins from selected species. A heatmap is presented to visualize the protein sequence similarity (lower left) and identity (upper right) values for R-RAS2-like and R-RAS2 proteins from selected organisms, generated using Morpheus. Warm colors (yellow and red) indicate high amino acid similarity (> 50%), while blue represents low similarity (< 50%). The protein sequence accession numbers and the corresponding identity/similarity percentages matrices (calculated using MatGAT2.01 with Matrix BLOSUM62 scores, can be found in Additional file 3 and 4, respectively). **Figure S3.** Sponge and human R-RAS2 homologs have similar but not identical localization in membranes of HeLa cells. Colocalization (yellow) of human R-RAS2 (green) with sponge homolog EsuRRAS2L (red) in the membranes of HeLa cells. Cells were seeded at density 2 × 10^4^ and transfected using Lipofectamine 3000. Human R-RAS2 was fluorescently labelled with GFP, and sponge EsuRRAS2L was fluorescently labelled with CHERRY. Twenty-four hours upon transfection, cells were fixed with paraformaldehyde and nuclei were stain using Hoechst. Cells were analyzed by confocal microscopy. The experiments were repeated three times in biological duplicates. Esu-sponge *Eunapius subterraneus*, Hsa-human. Scale bar – 10 μm. **Figure S4.** EsuRRAS2L partially colocalizes with RAB11. A) Colocalization (yellow) of sponge EsuRRAS2L or human HsaRRAS2 fluorescently labelled with GFP (green) with RAB11 (marker for recycling endosomes, red). B) Quantification of colocalization between EsuRRAS2L or HsaRRAS2 with RAB11 was done using ImageJ Coloc2 plugin and shown as Pearson’s correlation coefficient. *** p < 0.001, n = 30 cells per group from three different experiments. Hoechst was used to stain nuclei. Cells were analyzed by confocal microscopy. Levels of overexpressed C) HsaRRAS2 or D) EsuRRAS2L labelled with FLAG, and endogenous levels of RAB11 were analyzed by Western blot and detected with specific primary antibodies. E) and F) Quantification of RAB11 protein levels was shown as ratio to empty-vector control. Amido Black was used as a loading control. Cropped blots are displayed. Esu-sponge *Eunapius subterraneus*, Hsa-human. Scale bar – 10 μm. **Figure S5.** Both sponge and human R-RAS2 homologs alter the appearance of F-actin in MDA-MB-435S cells (ATCC cat. no. HTB-129). (A) Cells were seeded at density 4 × 10^4^ and transfected using Lipofectamine 2000. Forty-eight hours upon transfection with either empty vector of vector containing EsuRRAS2-like-GFP or HsaRRAS2-GFP construct, cells were fixed with paraformaldehyde and incubated with Alexa-Flour 488 conjugated phalloidin for F-actin visualization and anti-β5 antibody followed by Alexa-Fluor 647-conjugated secondary antibody and IRM images were taken. Analysis was performed using TCS SP8 Leica. Scale bar – 10 μm. (B) Quantification of data presented in (A). Scatter plots with median marked in red represent measurements of size and number of integrin β5 structures and measurements of percentage of actin fibers area per cell (n = 2). Data were analysed by one-way analysis of variance (ANOVA) with Dunnett’s multiple comparison. * P < 0.05. Esu-sponge *Eunapius subterraneus*, Hsa-human. **Table S1.** List of primers and constructs used in the study. **Table S2.** Amounts and corresponding concentrations of protein HsaRRAS2 used for GTPase activity assay. **Table S3.** Amounts and corresponding concentrations of protein EsuRRAS2-like used for GTPase activity assay. **Table S4.** List of antibodies and stains used in this study.

**Additional file 3. **The amino acid sequence identity and similarity percentages of R-RAS2-like proteins from sponges and R-RAS, R-RAS2, and M-RAS from human.

**Additional file 4. **The amino acid sequence identity and similarity percentages of R-RAS2 and R-RAS2-like homologs from selected species.

**Additional file 5. **Original data of western blots.

## Data Availability

All the data generated or analyzed during this study are included in this published article and its supplementary information files.

## Abbreviations

BRAF: V-raf murine sarcoma viral oncogene homolog B1
CLL: Chronic lymphocytic leukemia
DTBP: Dimethyl 3,3'-dithiobispropionimidate (Wang and Richard’s reagent)
EEA1: Early endosome antigen 1
F-actin: Filamentous actin
GDP: Guanosine diphosphate
GFP: Green fluorescent protein
GTP: Guanosine triphosphate
HVR: Hypervariable regions
IRM: Interference reflection microscopy
LAMP1: Lysosomal-associated membrane protein 1
MTT: 3-(4,5-Dimethylthiazol-2-yl)-2,5-diphenyltetrazolium bromide
NF1: Neurofibromatosis type 1 gene
PI3K: Phosphoinositide 3-kinase
poly(U): Polyuridylic acid
Rab5: Ras-related protein 5
Rab7: Ras-related protein 7
R-RAS: Ras-related protein
*r-ras*: Ras-related gene
R-RAS2: Ras-related 2 protein
*r-ras2*: Ras-related 2 gene
SNARE: Soluble N-ethylmaleimide attachment protein receptor
TfR: Transferrin receptor
