# Supplementary material for: The ancestral type of the R-RAS protein has oncogenic potential
Source: Cell Mol Biol Lett. 2024 Feb 21;29:27. doi: 10.1186/s11658-024-00546-0 (PMC10882905; doi:10.1186/s11658-024-00546-0)
Supplement: Supplementary file 2 — Additional file 2: Figure S1. Multiple sequence analysis of R-RAS2 and R-RAS2-like homologs from selected species. The conserved regions are indicated above the alignment, starting with the N-terminus: G1 motif, SwitchI region, G2 motif, G3 motif, SwitchII region, G4 motif, G5 motif, hypervariable region (HVR), CaaX box. The R-RAS2 and R-RAS2-like amino acid sequences were aligned using the ClustalX 2.0, and the alignment was visualised using the GeneDoc v2.7. Similar amino acids conserved among the analyzed proteins at levels of 100%, 80%, and 60% are represented as white letters on black background, white letters on dark grey background, and black letters on light grey background, respectively. Figure S2. Heatmap of amino acid sequence similarity and identity for R-RAS2-like and R-RAS2 proteins from selected species. A heatmap is presented to visualize the protein sequence similarity (lower left) and identity (upper right) values for R-RAS2-like and R-RAS2 proteins from selected organisms, generated using Morpheus. Warm colors (yellow and red) indicate high amino acid similarity (> 50%), while blue represents low similarity (< 50%). The protein sequence accession numbers and the corresponding identity/similarity percentages matrices (calculated using MatGAT2.01 with Matrix BLOSUM62 scores, can be found in Additional file 3 and 4, respectively). Figure S3. Sponge and human R-RAS2 homologs have similar but not identical localization in membranes of HeLa cells. Colocalization (yellow) of human R-RAS2 (green) with sponge homolog EsuRRAS2L (red) in the membranes of HeLa cells. Cells were seeded at density 2 × 10^4^ and transfected using Lipofectamine 3000. Human R-RAS2 was fluorescently labelled with GFP, and sponge EsuRRAS2L was fluorescently labelled with CHERRY. Twenty-four hours upon transfection, cells were fixed with paraformaldehyde and nuclei were stain using Hoechst. Cells were analyzed by confocal microscopy. The experiments were repeated three times in biologi [file 11658_2024_546_MOESM2_ESM.docx]

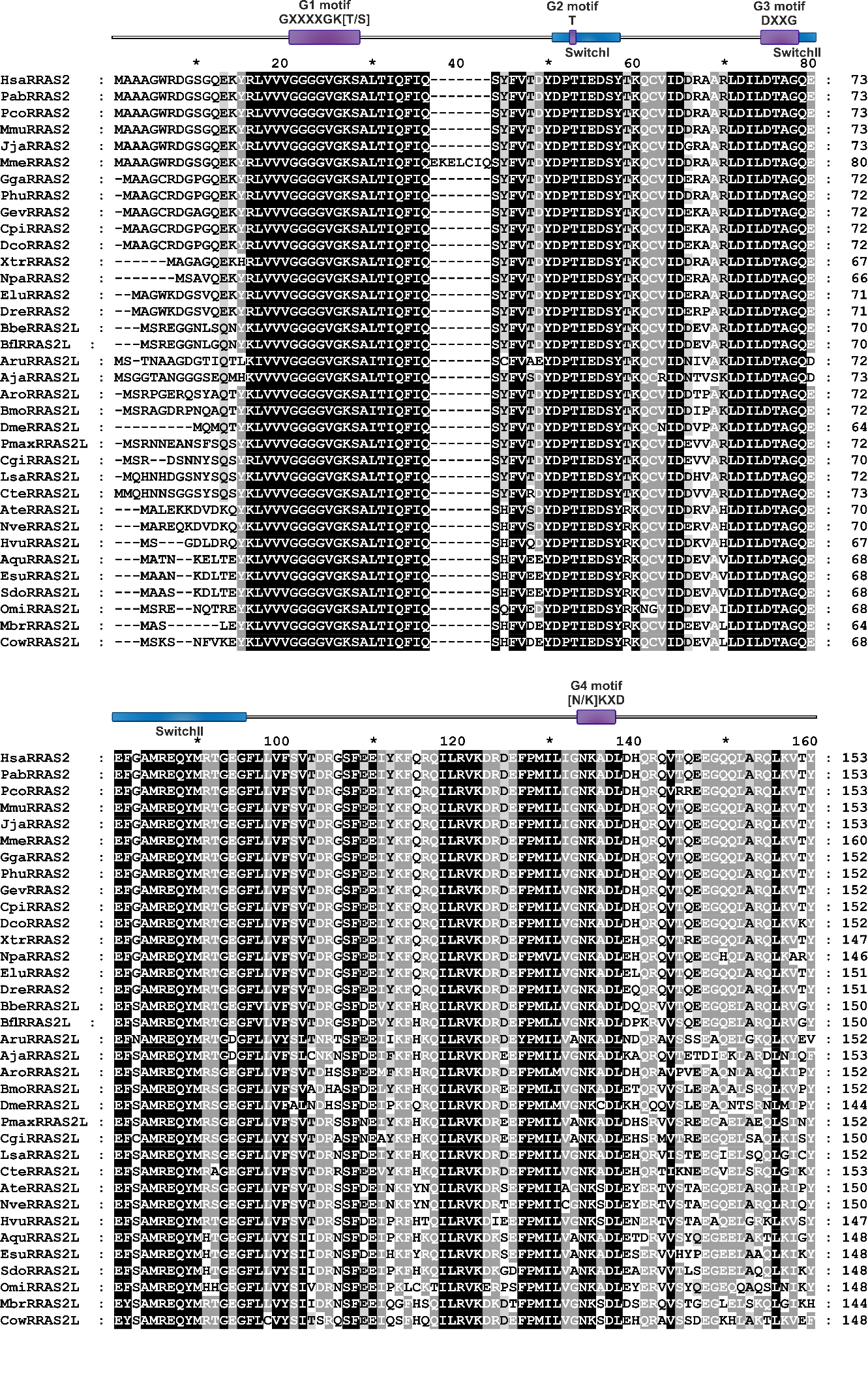

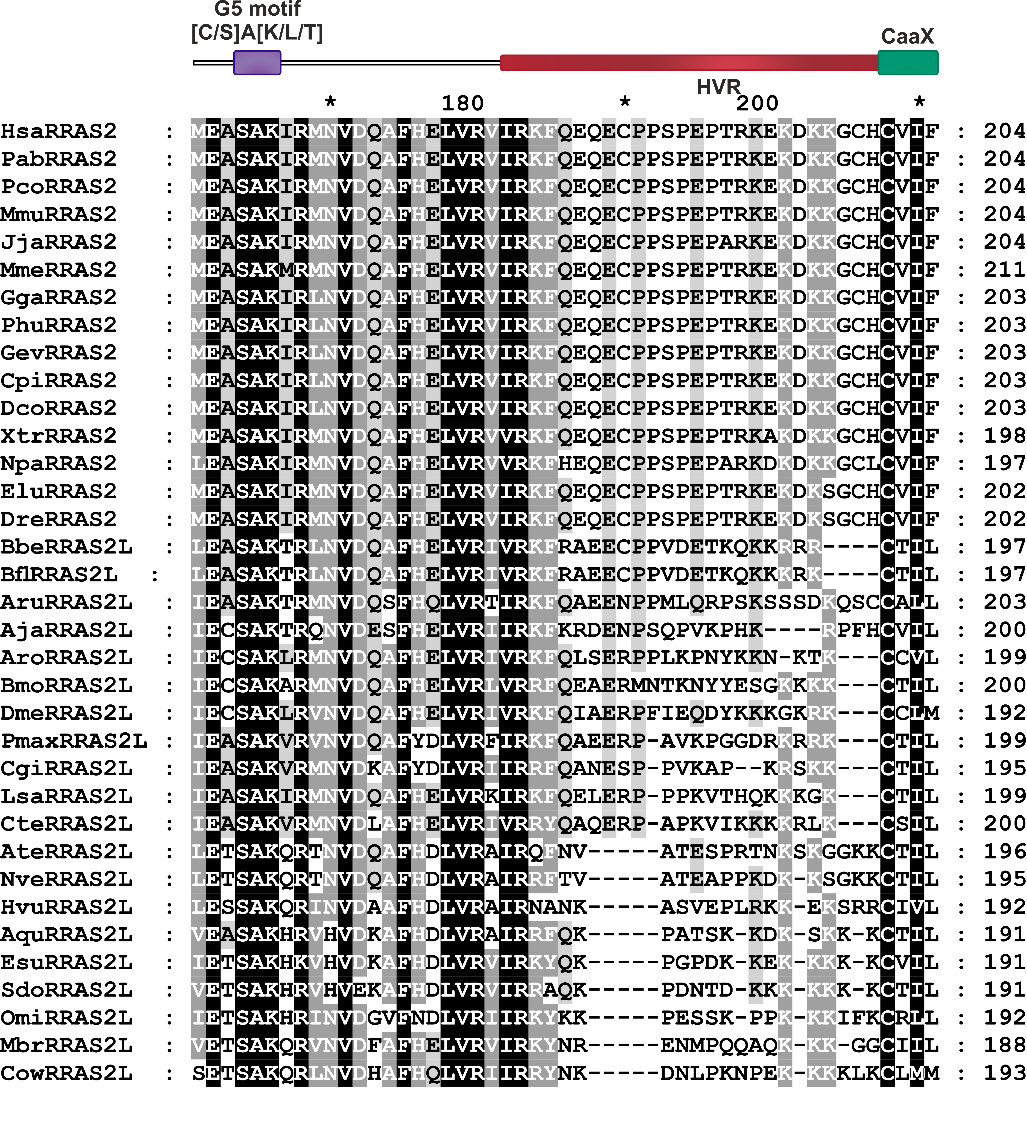


**Figure S1. Multiple sequence analysis of R-RAS2 and R-RAS2-like homologs from selected species.** The conserved regions are indicated above the alignment, starting with the N-terminus: G1 motif, *SwitchI* region, G2 motif, G3 motif, *SwitchII* region, G4 motif, G5 motif, hypervariable region (HVR), CaaX box. The R-RAS2 and R-RAS2-like amino acid sequences were aligned using the ClustalX 2.0, and the alignment was visualised using the GeneDoc v2.7. Similar amino acids conserved among the analyzed proteins at levels of 100%, 80%, and 60% are represented as white letters on black background, white letters on dark grey background, and black letters on light grey background, respectively.


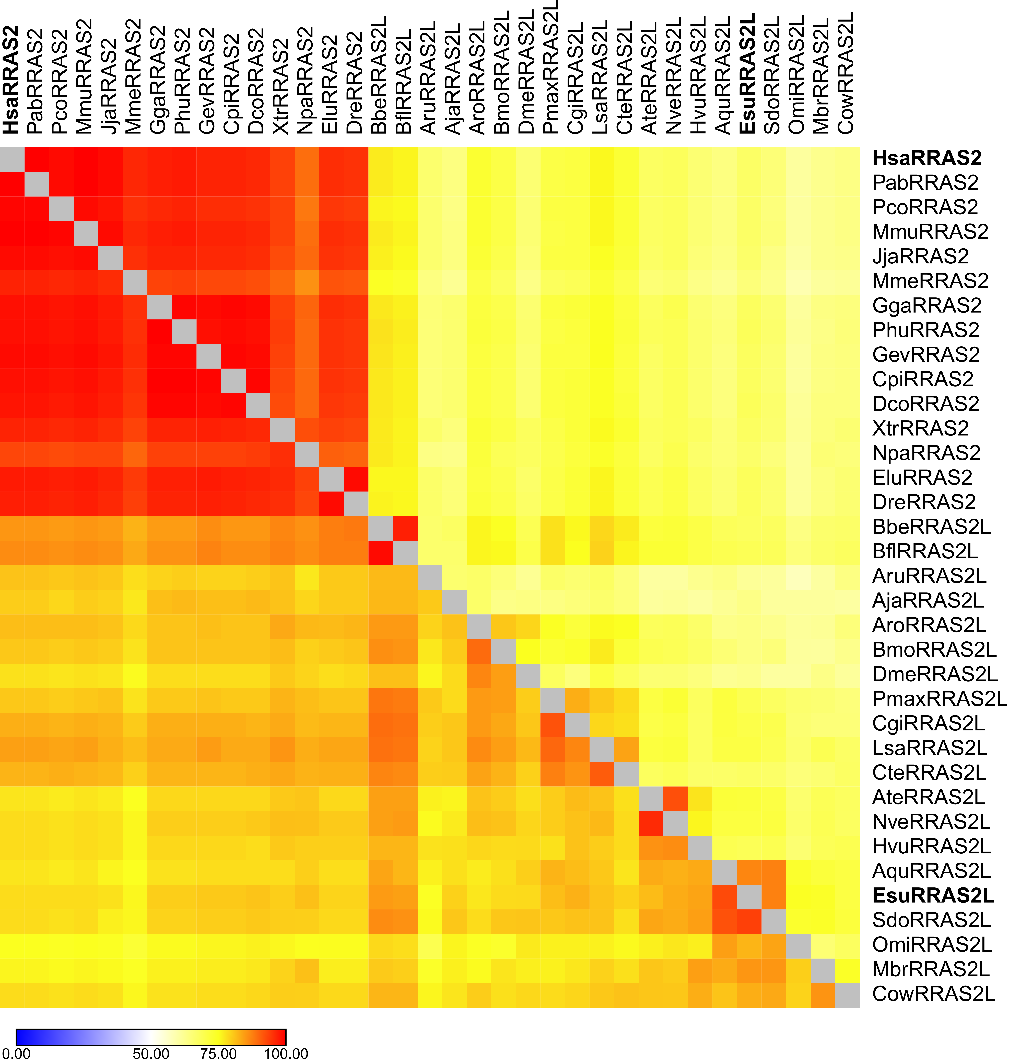


**Figure S2. Heatmap of amino acid sequence similarity and identity for R-RAS2-like and R-RAS2 proteins from selected species.** A heatmap is presented to visualize the protein sequence similarity (lower left) and identity (upper right) values for R-RAS2-like and R-RAS2 proteins from selected organisms, generated using Morpheus. Warm colors (yellow and red) indicate high amino acid similarity (>50%), while blue represents low similarity (<50%). The protein sequence accession numbers and the corresponding identity/similarity percentages matrices (calculated using MatGAT2.01 with Matrix BLOSUM62 scores, can be found in Additional file 3 and 4, respectively).


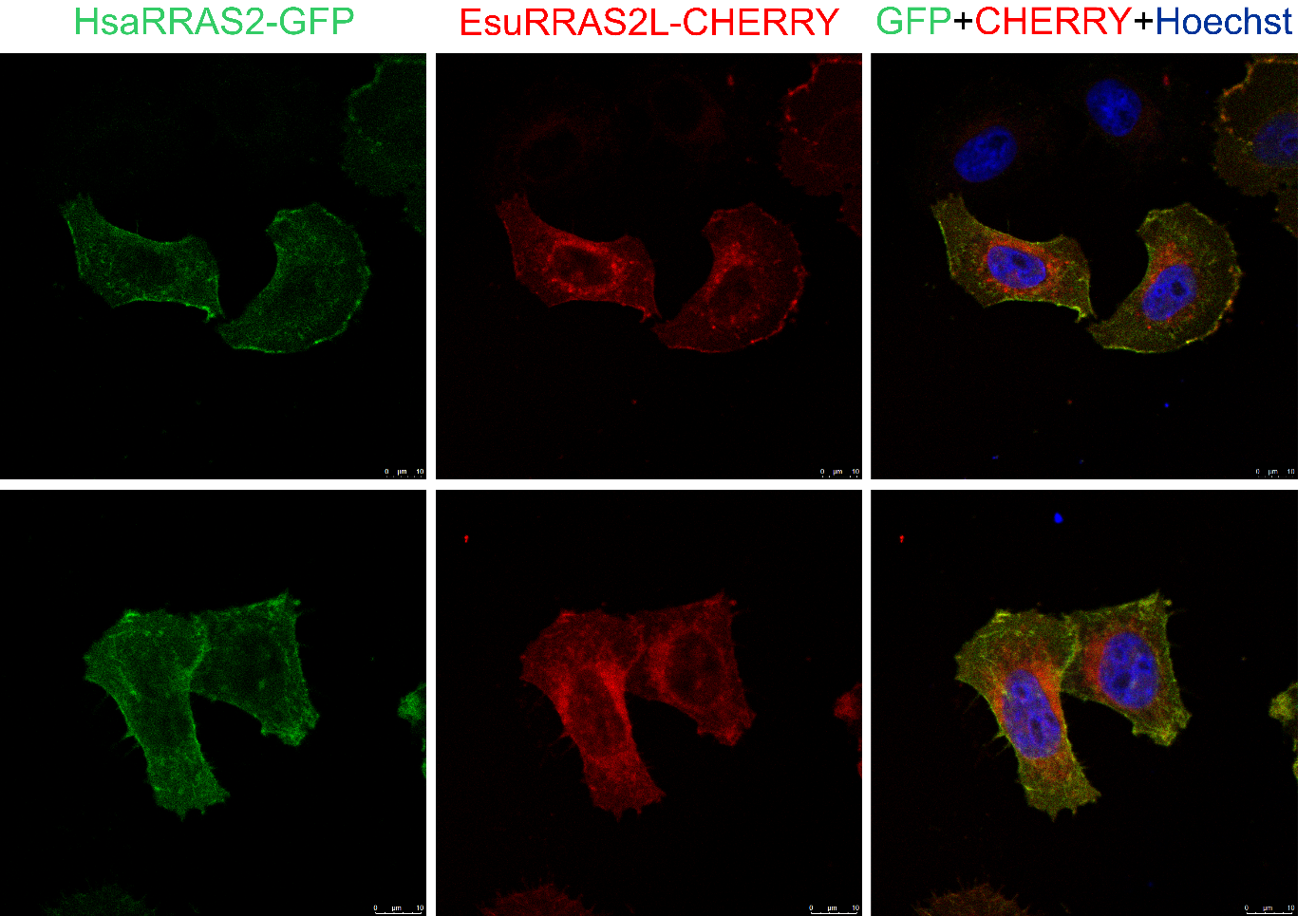


**Figure S3.** **Sponge and human R-RAS2 homologs have similar but not identical localization in membranes of HeLa cells**. Colocalization (yellow) of human R-RAS2 (green) with sponge homolog EsuRRAS2L (red) in the membranes of HeLa cells. Cells were seeded at density 2×10^4^ and transfected using Lipofectamine 3000. Human R-RAS2 was fluorescently labelled with GFP, and sponge EsuRRAS2L was fluorescently labelled with CHERRY. Twenty-four hours upon transfection, cells were fixed with paraformaldehyde and nuclei were stain using Hoechst. Cells were analyzed by confocal microscopy. The experiments were repeated three times in biological duplicates. Esu-sponge *Eunapius subterraneus*, Hsa-human. Scale bar – 10 μm.


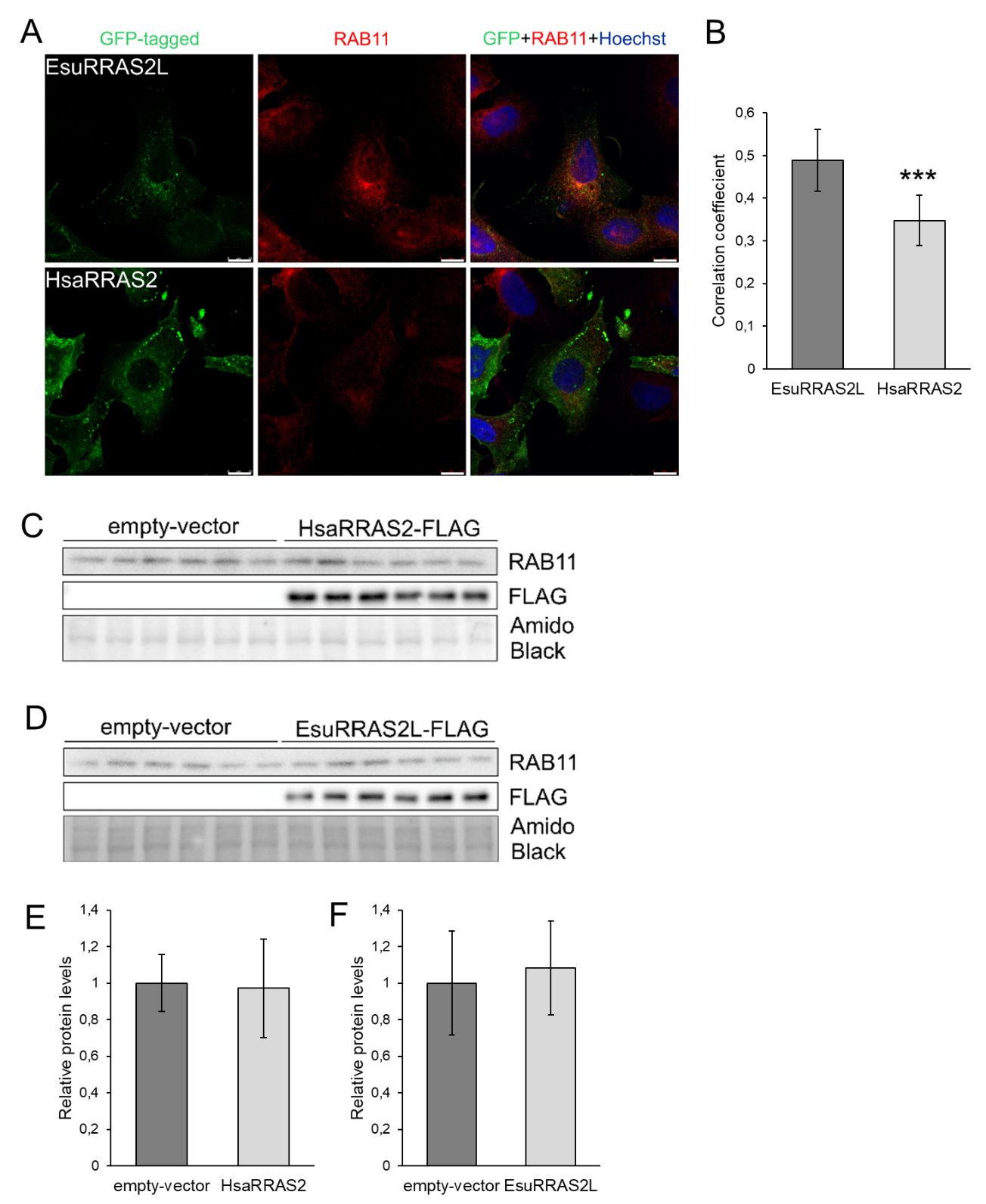


**Figure S4. EsuRRAS2L partially colocalizes with RAB11.** A) Colocalization (yellow) of sponge EsuRRAS2L or human HsaRRAS2 fluorescently labelled with GFP (green) with RAB11 (marker for recycling endosomes, red). B) Quantification of colocalization between EsuRRAS2L or HsaRRAS2 with RAB11 was done using ImageJ Coloc2 plugin and shown as Pearson’s correlation coefficient. *** p < 0.001, n = 30 cells per group from three different experiments. Hoechst was used to stain nuclei. Cells were analyzed by confocal microscopy. Levels of overexpressed C) HsaRRAS2 or D) EsuRRAS2L labelled with FLAG, and endogenous levels of RAB11 were analyzed by Western blot and detected with specific primary antibodies. E) and F) Quantification of RAB11 protein levels was shown as ratio to empty-vector control. Amido Black was used as a loading control. Cropped blots are displayed. Esu-sponge *Eunapius subterraneus*, Hsa-human. Scale bar – 10 μm.


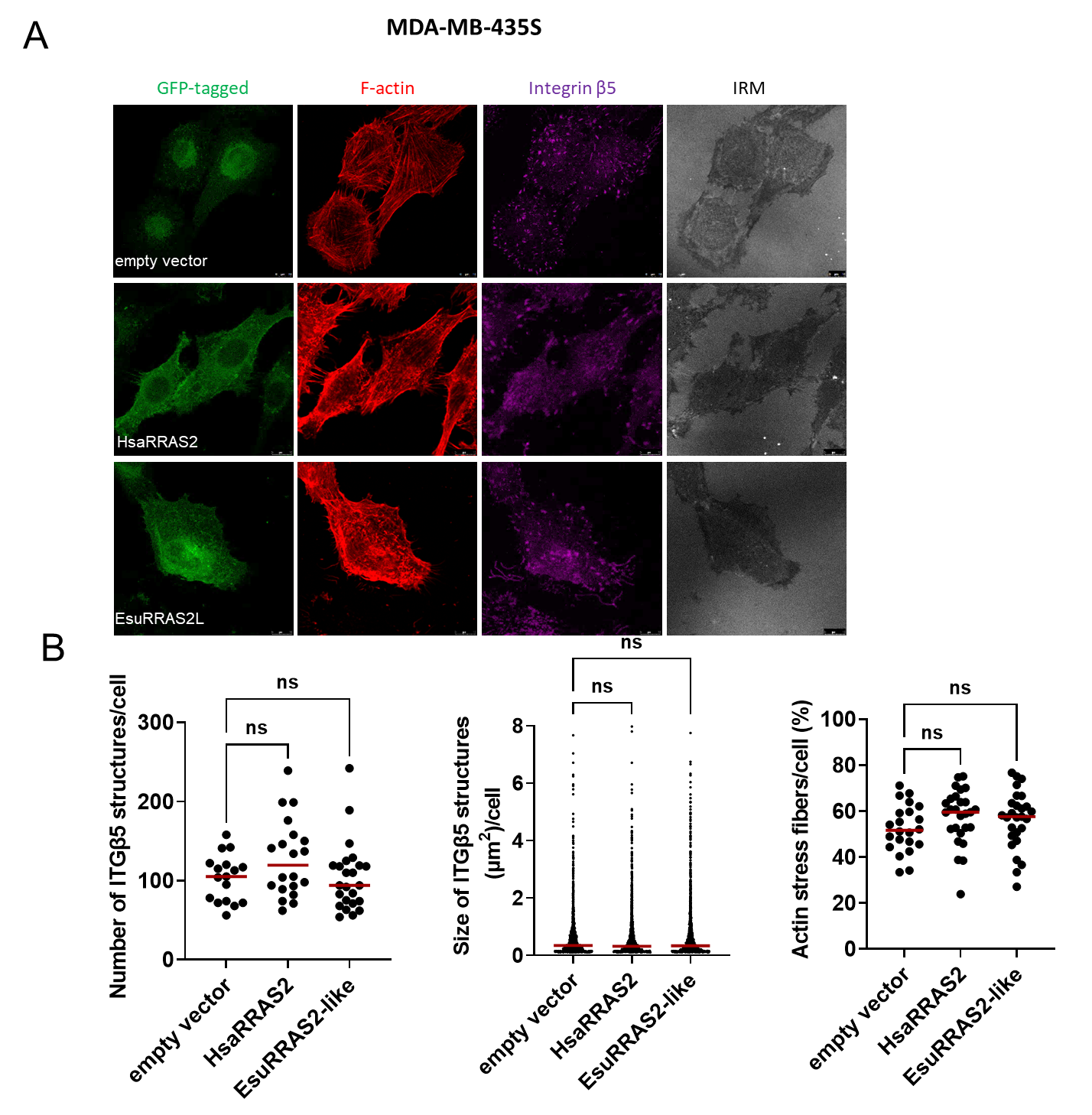


**Figure S5. Both sponge and human RRAS2 homologs alter the appearance of F-actin in MDA-MB-435S cells** (ATCC cat. no. HTB-129). (A) Cells were seeded at density 4×10^4^ and transfected using Lipofectamine 2000. Forty-eight hours upon transfection with either empty vector of vector containing EsuRRAS2-like-GFP or HsaRRAS2-GFP construct, cells were fixed with paraformaldehyde and incubated with Alexa-Flour 488 conjugated phalloidin for F-actin visualization and anti-β5 antibody followed by Alexa-Fluor 647-conjugated secondary antibody and IRM images were taken. Analysis was performed using TCS SP8 Leica. Scale bar – 10 μm. (B) Quantification of data presented in (A). Scatter plots with median marked in red represent measurements of size and number of integrin β5 structures and measurements of percentage of actin fibers area per cell (n=2). Data were analysed by one-way analysis of variance (ANOVA) with Dunnett’s multiple comparison. * P < 0.05. Esu-sponge *Eunapius subterraneus*, Hsa-human.

**Table S1. List of primers and constructs used in the study.**

| **Construct Name/ Organism** | **Origin** | **Cloned in/ TAG** | **Primers/restriction site** |
| --- | --- | --- | --- |
| EsuRRAS2-like_pET28b  *E. subterraneus* | Esu cDNA | / | 5'-ACGATGGCGGCCAACAAAGACCTAAC-3'  5'-GGAGCTCACAGAATTACACATTTCTTC-3' |
| EsuRRAS2-like_pET28b  *E. subterraneus* | Esu cDNA | pEt28b  6xHis (N ter) | NdeI: 5'-GTCTAGCATATGGCGGCCAACAAAGACCTAACCG-3'  BamHI: 5'-CTAGACGGATCCTCACAGAATTACACATTTCTTC-3' |
| HsaRRAS2_pET28b  *H. sapiens* | RC204591  OriGene | pET28b  6xHis (N ter) | NdeI: 5'-GTCTAGCATATGGCCGCGGCCGGCTGGCG-3'  XhoI: 5'-CTAGACCTCGAGTTAGAAAATGACACAATGGCAG-3' |
| EsuRRAS2-like_pEGFP-C1  *E. subterraneus* | Esu cDNA | pEGFP-C1  GFP (N ter) | XhoI: 5'-GTCTAGCTCGAGGCATGGCGGCCAACAAAGAC-3'  BamHI: 5'-CTAGACGGATCCTCACAGAATTACACATTTCTTC-3' |
| HsaRRAS2_pEGFP-C1  *H. sapiens* | RC204591  OriGene | pEGFP-C1  GFP (N ter) | XhoI: 5'-GTCTAGCTCGAGGCATGGCCGCGGCCGGCTGGCG-3'  BamHI: 5'-CTAGACGGATCCTTAGAAAATGACACAATGGC-3' |
| EsuRRAS2-like_pmCherry-C1  *E. subterraneus* | Esu cDNA | pmCherry-C1  CHERRY (N ter) | XhoI: 5'-GTCTAGCTCGAGGCATGGCGGCCAACAAAGACCTAACCG-3'  BamHI: 5'-CTAGACGGATCCTCACAGAATTACACATTTCTTC-3' |
| HsaRRAS2_pmCherry-C1  *H. sapiens* | RC204591  OriGene | pmCherry-C1  CHERRY (N ter) | XhoI: 5'-GTCTAGCTCGAGGCATGGCCGCGGCCGGCTGGCG-3'  BamHI: 5'-CTAGACGGATCCTTAGAAAATGACACAATGGCAG-3' |
| EsuRRAS2-like-FLAG  *E. subterraneus* | Esu cDNA | pcDNA3.1  FLAG (N ter) | EcoRI: 5'-GTCTAGGAATTCACGAGATGGACTACAAGGACGACGACGATAAGATGGCGGCCAACAAAGAC-3'  XhoI: 5'-CTAGACCTCGAGTCACAGAATTACACATTTCTTC-3' |
| HsaRRAS2-FLAG  *H. sapiens* | RC204591  OriGene | pcDNA3.1  FLAG (N ter) | EcoRI: 5'-GTCTAGGAATTCATGGACTACAAGGACGACGACGATAAGATGGCCGCGGCCGGCTGGCGG-3'  XhoI: 5'-CTAGACCTCGAGTTAGAAAATGACACAATGGCAG-3' |

**Table S2. Amounts and corresponding concentrations of protein HsaRRAS2 used for GTPase activity assay.**

| **HsaRRAS2 (ng)** | **Log_10_ HsaRRAS2 Amount (ng)** | **HsaRRAS2 Concentration (µM)** | **Luminescence (RLU)** |
| --- | --- | --- | --- |
| 6335.000 | 3.802 | 50.000 | 5982.333 |
| 3167.500 | 3.501 | 25.000 | 9466.000 |
| 1583.750 | 3.200 | 12.500 | 79588.500 |
| 791.875 | 2.899 | 6.250 | 230109.000 |
| 395.938 | 2.598 | 3.125 | 313989.000 |
| 197.969 | 2.297 | 1.563 | 332440.000 |
| 98.984 | 1.996 | 0.781 | 369244.333 |
| 49.492 | 1.695 | 0.391 | 374240.333 |
| 24.746 | 1.394 | 0.195 | 358654.667 |
| 12.373 | 1.092 | 0.098 | 364338.000 |
| 6.187 | 0.791 | 0.049 | 382041.000 |
| / | / | / | 416282.333 |

**Table S3. Amounts and corresponding concentrations of protein EsuRRAS2-like used for GTPase activity assay.**

| **EsuRRAS2-like (ng)** | **Log_10_ EsuRRAS2-like Amount (ng)** | **EsuRRAS2-like Concentration (µM)** | **Luminescence (RLU)** |
| --- | --- | --- | --- |
| 6335.000 | 3.802 | 50.000 | 4056.667 |
| 3167.500 | 3.501 | 25.000 | 12563.833 |
| 1583.750 | 3.200 | 12.500 | 52549.667 |
| 791.875 | 2.899 | 6.250 | 266606.000 |
| 395.938 | 2.598 | 3.125 | 376491.000 |
| 197.969 | 2.297 | 1.563 | 417307.667 |
| 98.984 | 1.996 | 0.781 | 440374.833 |
| 49.492 | 1.695 | 0.391 | 440237.667 |
| 24.746 | 1.394 | 0.195 | 462968.667 |
| 12.373 | 1.092 | 0.098 | 473606.833 |
| 6.187 | 0.791 | 0.049 | 503627.000 |
| / | / | / | 529470.833 |

**Table S4. List of antibodies and stains used in this study.**

| **Primary antibodies** | **Cat. no.** | **Distributor** | **species** | **ICC** | **WB** |
| --- | --- | --- | --- | --- | --- |
| FLAG Tag (DYKDDDDK Tag, D6W5B) | #14793 | Cell Signaling, USA | rabbit | / | 1:1000 |
| Early endosome antigen 1 (EEA1) | #610457 | Cell Signaling Technology, USA | mouse | 1:250 | 1:1000 |
| Transferrin receptor (TfR), clone H68.4 | 136800 | Life Technologies, USA | mouse | 1:100 | 1:1000 |
| Rab7 | ab137029 | Abcam, UK | rabbit | 1:100 | 1:1000 |
| Lysosomal associated membrane protein (LAMP1) | L1418 | Sigma, USA | rabbit | 1:100 | 1:1000 |
| Integrin β5 | D24A5 | Cell Signaling, USA | rabbit | 1:600 | 1:1000 |
| Talin 1, clone 97H6 | MCA4770GA | Bio-Rad, UK | mouse | / | 1:1000 |
| Vinculin, clone EPR8185 | ab129002 | Abcam, UK | rabbit | / | 1:10000 |
| RAB11 | D4F5 | Cell Signaling, USA | rabbit | 1:100 | 1:2000 |
|  | | | | | |
| **Secondary antibodies** | **Cat. no.** | **Distributor** | **species** | **ICC** | **WB** |
| Donkey anti-Rabbit IgG (H+L) Highly Cross-Adsorbed Secondary Antibody, Alexa Fluor™ 594 | A21207 | Thermo Fisher Scientific, USA | donkey | 1:500 | / |
| Donkey anti-Mouse IgG (H+L) Highly Cross-Adsorbed Secondary Antibody, Alexa Fluor™ 594 | A21203 | Thermo Fisher Scientific, USA | donkey | 1:500 | / |
| Goat anti-Rabbit IgG (H+L)  [F(ab')2 Fragment, Alexa Fluor® 647 Conjugate](https://www.cellsignal.com/products/secondary-antibodies/anti-rabbit-igg-h-l-f-ab-2-fragment-alexa-fluor-647-conjugate/4414) | 4414 | Cell Signaling, USA | goat | 1:1000 | / |
| Goat Anti-Rabbit IgG (H + L)-HRP Conjugate | 1706515 | Bio-Rad, UK | goat | / | 1:5000 |
| Goat Anti-Mouse IgG (H + L)-HRP Conjugate | 1706516 | Bio-Rad, UK | goat | / | 1:5000 |
|  | | | | | |
| **Stain** | **Cat. no.** | **Distributor** | **species** | **ICC** | **WB** |
| Alexa-Flour 555 conjugated phalloidin | #8953 | Cell Signaling Technology, USA | / | 1:40 | / |
| Alexa-Flour 488 conjugated phalloidin | P5282 | Sigma-Aldrich, USA | / | 1:100 | / |
| Hoechst 33342 | H3570 | Sigma-Aldrich, USA | / | 1:200 | / |
