# Supplementary material for: The ancestral type of the R-RAS protein has oncogenic potential
Source: Cell Mol Biol Lett. 2024 Feb 21;29:27. doi: 10.1186/s11658-024-00546-0 (PMC10882905; doi:10.1186/s11658-024-00546-0)
Supplement: Supplementary file 5 — Additional file 5. Original data of western blots. [file 11658_2024_546_MOESM5_ESM.docx]

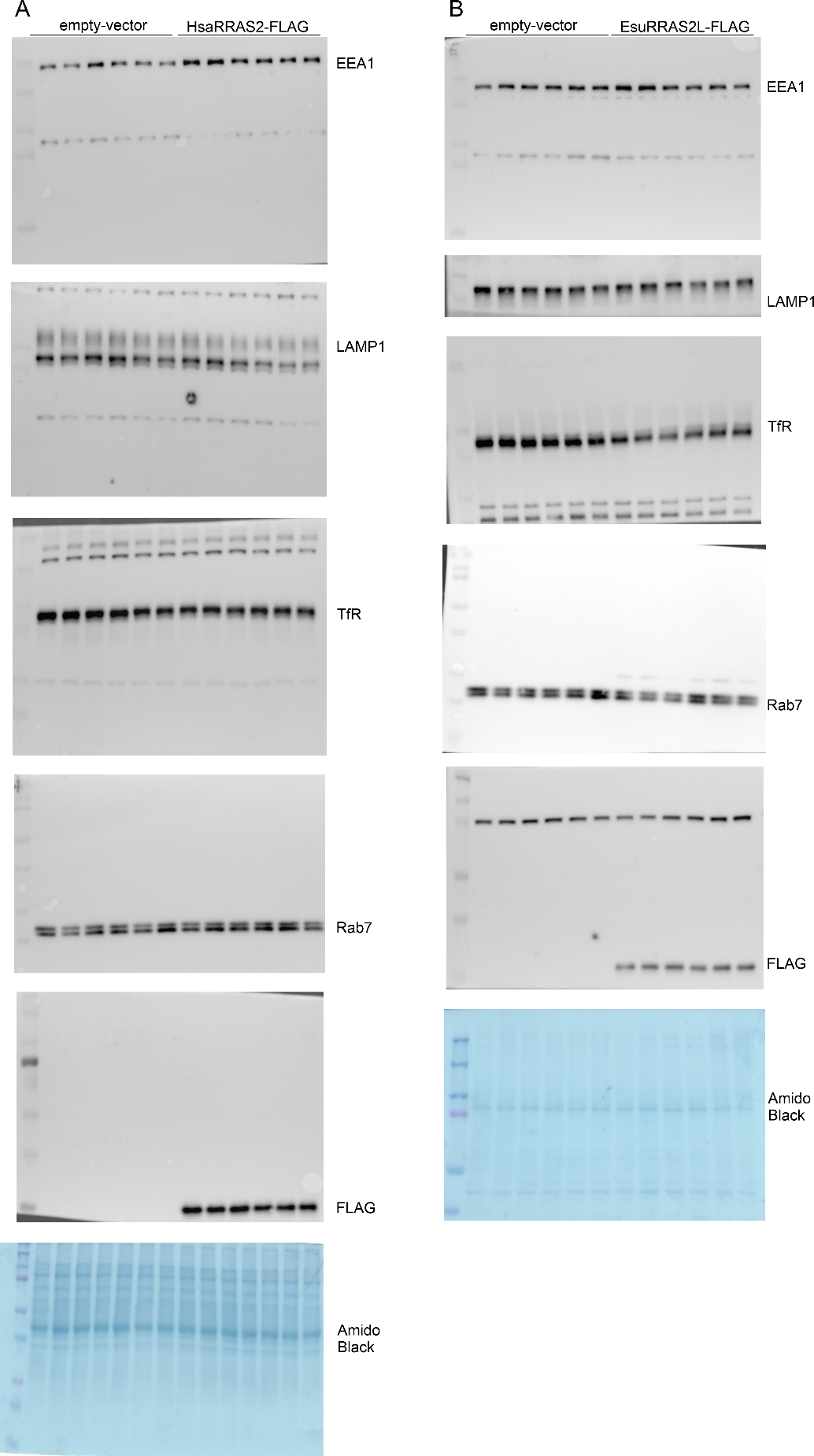


Figure 6. Original data of western blots


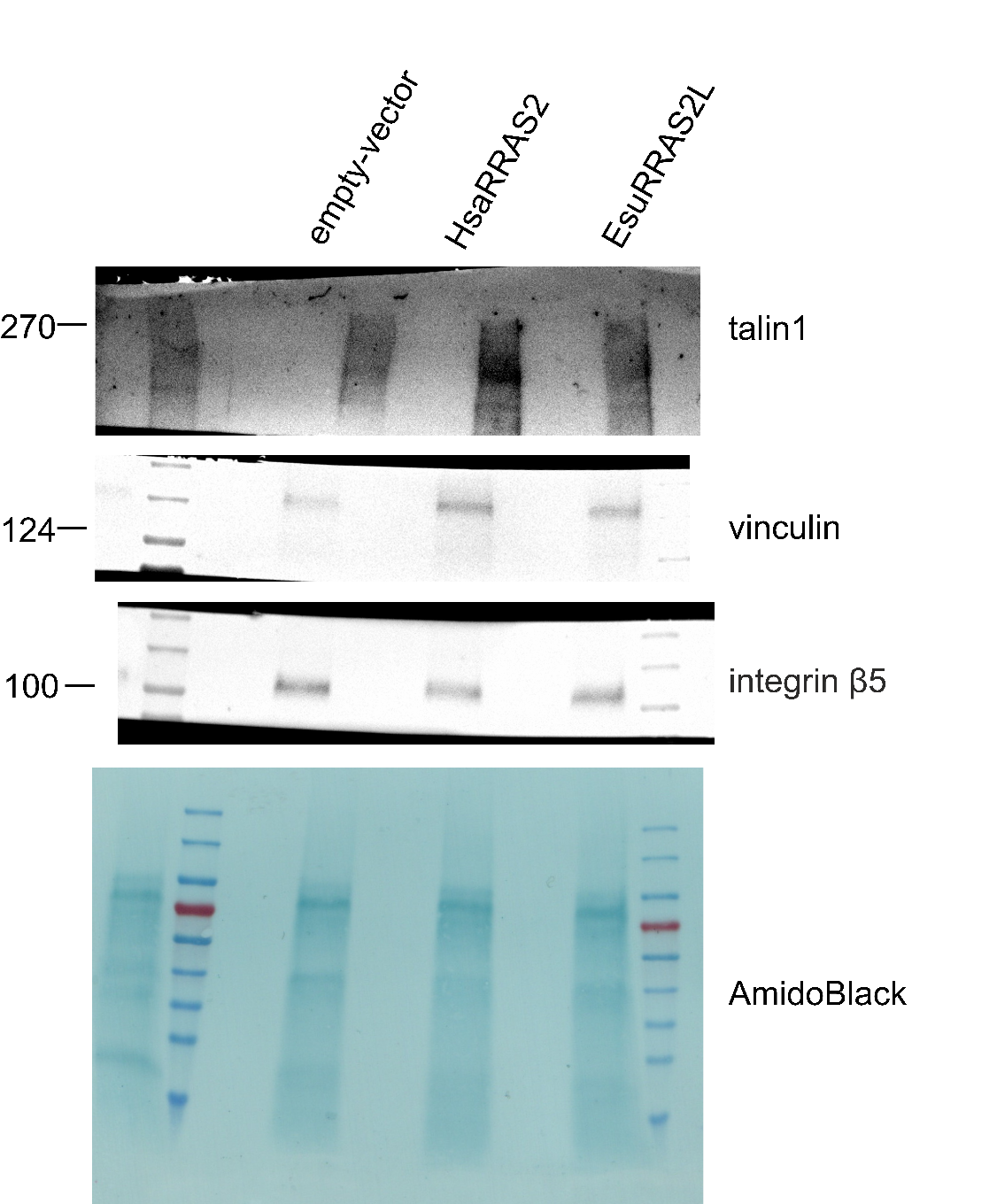


Figure 8. Original data of western blots


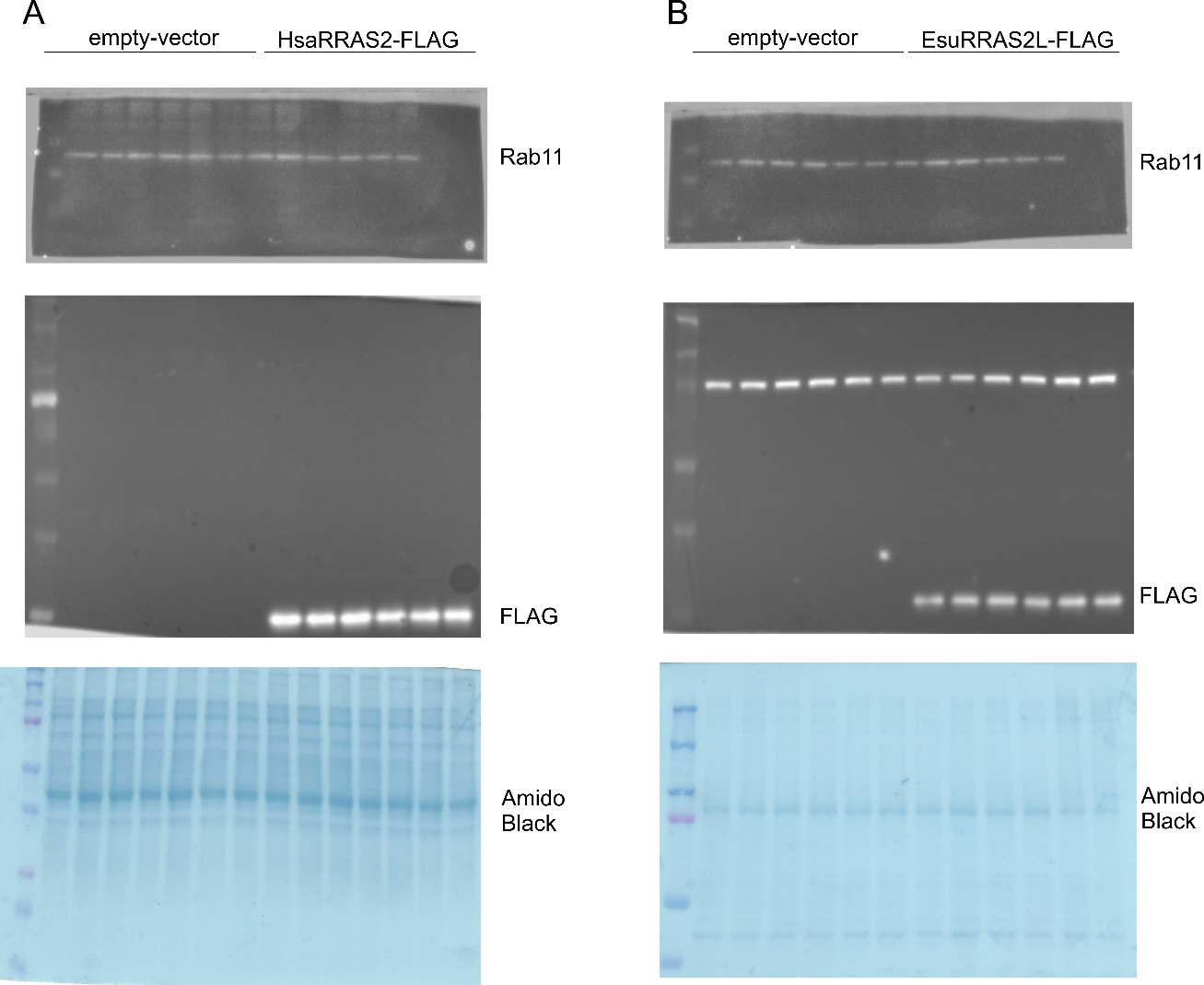


Fig S4. Original data of western blots
